# Customizing Tactile Sensors via Machine Learning‐Driven Inverse Design

**DOI:** 10.1002/advs.202524250

**Published:** 2026-01-28

**Authors:** Baocheng Wang, Depeng Kong, Zhiao He, Jikai Liang, Yuyao Lu, Zikang Deng, Honghe Li, Mengke Wang, M. Jamal Deen, Zhiqiu Ye, Shuyao Zhou, Huayong Yang, Honghao Lyu, Jun Chen, Kaichen Xu, Geng Yang

**Affiliations:** ^1^ State Key Laboratory of Fluid Power and Mechatronic Systems, School of Mechanical Engineering Zhejiang University Hangzhou China; ^2^ Dongfang Electric (Hangzhou) Innovation Institute Co., Ltd. Hangzhou China; ^3^ ZJU‐UIUC Institute Zhejiang University Haining China; ^4^ Department of Electrical and Computer Engineering McMaster University Hamilton Ontario Canada; ^5^ Institute of AI for Industries Chinese Academy of Sciences Nanjing China; ^6^ Zhejiang Key Laboratory of Intelligent Robot for Operation and Maintenance Hangzhou China; ^7^ Department of Bioengineering University of California, Los Angeles Los Angeles California USA

**Keywords:** inverse design, machine learning, microstructure, tactile sensor

## Abstract

Replicating the sophisticated sense of touch in artificial systems requires tactile sensors with precisely tailored properties. However, manually navigating the complex microstructure‐property relationship results in inefficient and suboptimal designs. Here, we present a machine learning‐accelerated, multi‐objective inverse design methodology for the automatic customization of tactile sensors. At its core is a data‐efficient microstructure‐property predictor designed to ensure robust accuracy with minimal experimental data. It achieves this by synergistically combining support vector machine‐based boundary definition with dual‐phase active learning. This predictor then drives a multi‐objective inverse design software, enabling real‐time, on‐demand sensor customization. This methodology not only dramatically enhances the design efficiency but also yields sensors with exceptional characteristics—high sensitivity (1.2 V/kPa), high linearity (*R* ^2^ = 0.999), and wide detection range (0–400 kPa). The resulting sensors are successfully applied to pulse monitoring, material identification, and robotic grasping. Furthermore, the underlying microstructure‐property mechanisms are elucidated using interpretable machine learning. This work establishes a general paradigm for automated sensor customization, accelerating the development of next‐generation wearable and robotic sensing systems.

## 1 Introduction

Inspired by the mechanoreceptors of human skin [1], tactile sensors, especially pressure sensors, have emerged as critical components in robot haptics [2, 3, 4], biomonitoring [5, 6], human–machine interfaces [7, 8, 9, 10], and metaverse [11, 12]. These pressure sensors detect pressure through converting mechanical stimuli into electric signals via triboelectric [13, 14], piezoresistive [15, 16], capacitive [17, 18], and magnetic [19, 20] transducing mechanisms. Their performance requirements vary significantly depending on specific applications. For instance, respiratory and pulse monitoring demand high sensitivity with low detection ranges (<1 kPa) to capture subtle signals [4]. In contrast, robotic manipulation applications necessitate exceptional linearity across broad detection ranges (0–300 kPa) to achieve precise force control [21]. However, such forward design approaches follow a structure‐to‐property workflow, struggling to address those diverse needs. The conventional design approach typically relies on simplified analytical models and trial‐and‐error tests to modulate microstructures for target performance [22, 23, 24]. This usually leads to substantial time costs and single‐objective regulation (e.g., linearity, sensitivity, detection range), particularly in high‐dimensional parameter spaces. These challenges are further exacerbated by the complex, nonlinear relationships among material properties, structural parameters, and sensor performance.

Inverse design, following a property‐to‐structure workflow, affords an efficient data‐driven strategy to resolve the dilemma in forward design strategies [25, 26, 27, 28]. Rather than relying on simplified analytical models, this approach uncovers the nonlinear relationships between design parameters and performance metrics by machine learning (ML) models. The inverse design has revolutionized fields such as mechanical metamaterial [29], composite material [30], drug discovery [31], and inorganic electrocatalysts [32]. Nevertheless, applying this methodology to device‐level pressure sensors has been impeded by data scarcity, stemming from the resource‐intensive nature of device testing [33, 34]. The data scarcity issue makes it difficult to develop accurate ML models for device‐level optimization of pressure sensors. One attempt explored the inverse design of high‐linearity pressure sensors using simulation‐driven surrogate models. However, this method remains limited by its dependence on single‐objective optimization within a fixed detection range (linearity improvement within 0–300 kPa) [35], falling short of meeting the diverse performance demands of real‐world applications.

**FIGURE 1:**
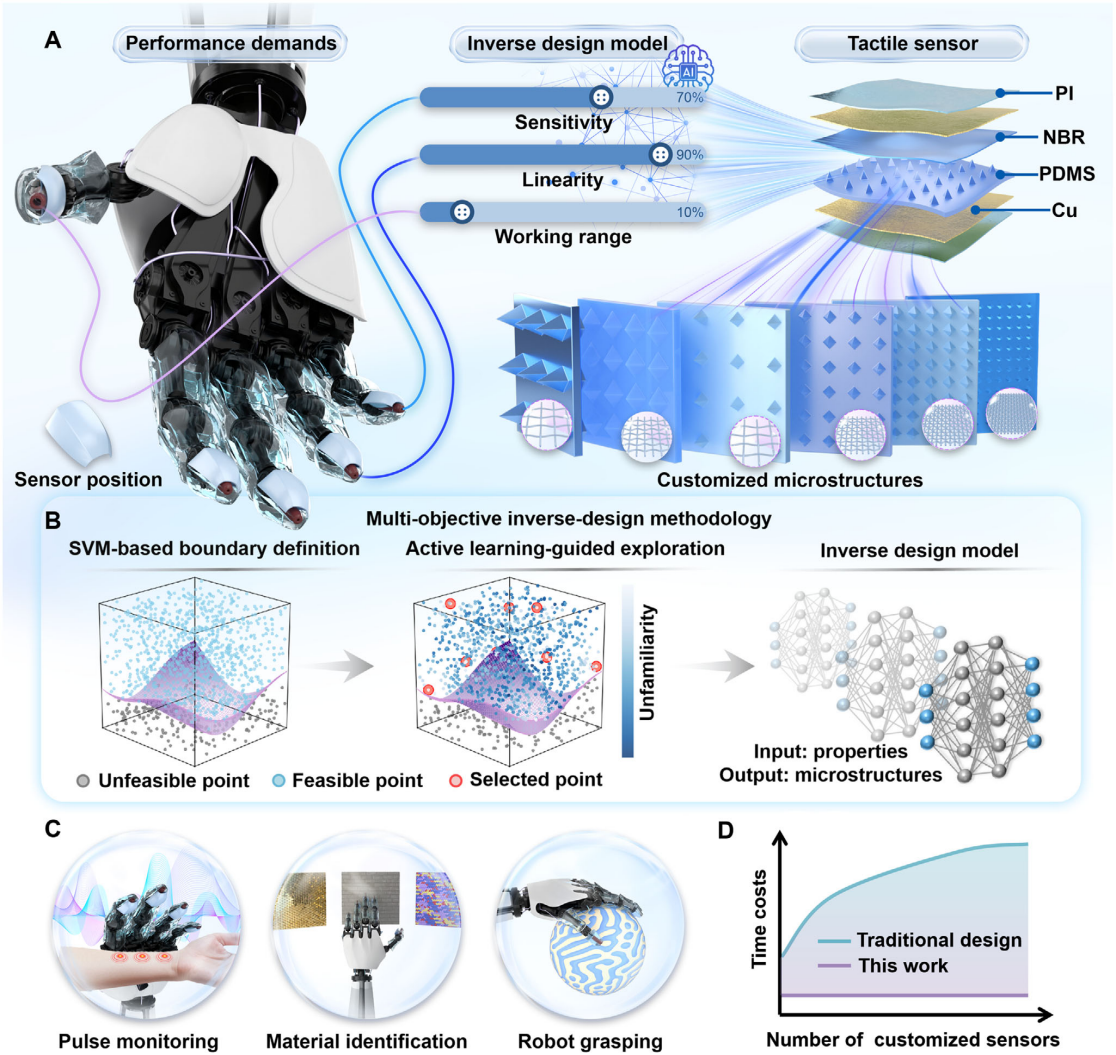
Machine learning‐enabled inverse design for the automatic customization of TPSs. (A) Inverse design model for customizing the sensitivity, linearity, and working range of tactile sensors. (B) Schematic illustration of the multi‐objective inverse design methodology powered by ML models. (C) Application scenarios of the inverse‐designed tactile sensors. (D) Schematic comparison of the time consumption trends as the number of customized sensors increases.

In this work, we create a multi‐objective inverse design methodology to accelerate the design of flexible pressure sensors with customizable sensitivity, linearity, and detection range (Figure 1A). The methodology consists of three meticulously designed steps (Figure 1B), validated by the inverse design of triboelectric pressure sensors (TPSs). First, we identify four critical microstructural design variables—crosslinker concentration, height, side length, and density—to establish a multidimensional design space for tunneling the sensor performance. This space is subsequently refined by a support‐vector machine (SVM), effectively filtering out poorly conformal parameter combinations. Second, a dual‐phase active learning (DP‐AL) algorithm efficiently navigates the design space, training a prediction model with only 90 experimentally validated sensors, which accurately predicts pressure‐voltage responses across 0–400 kPa. Third, in integration with a multi‐objective optimization algorithm, we propose an inverse design user interface (UI) that supports recommending optimal microstructures to satisfy the customized performance in seconds.

The customized sensors fulfill various performance demands of applications, including pulse monitoring, material identification, and robot grasping. Notably, compared with previously reported TPSs, the customized TPS first achieves a continuous linear response across 0–400 kPa, eliminating traditional nonlinear transition zones. Moreover, the contribution of different microstructure parameters on multiple sensor performance is quantified by a model interpretation algorithm, further validating the reliability of the methodology. This inverse design approach not only efficiently addresses the coupling of multiple performance metrics, but also establishes a customizable and automated methodology to meet the increasing demands of humanoid robotics and wearable electronics (Figure 1C). A comprehensive evaluation of design efficiency demonstrates the superiority of our inverse design approach over relevant forward and inverse design methodologies (Figure 1D; Note S1 and Table S1).

## 2 Results

### 2.1 Influential Microstructure Variables for Tuning Pressure Sensing Performance

A rational sensor design model is essential to the discovery of high‐performance TPSs. The design model of the flexible TPS is composed of two electrode layers (Cu), a positive triboelectric layer (nitrile rubber, NBR), a negative triboelectric layer (polydimethylsiloxane, PDMS) with customizable quadrangular pyramid, and two substrate layers (polyimide, PI), as illustrated in Figure 1A. PDMS‐NBR material pairing was selected owing to their widely separated positions in the triboelectric series [36], enabling a high surface charge density. The quadrangular pyramid microstructure configuration was adopted based on its demonstrated superiority in enhancing pressure sensing performance, as reported in previous works [37]. The working mechanism of the TPS is based on contact electrification and electrostatic induction, as illustrated in Figure S1 and Note S2. When external pressure is applied, the deformation of microstructures induces dynamic redistribution of surface charges, which is quantitatively translated into measurable output voltage signals, thereby enabling pressure sensing capabilities. It can be inferred that the sensing performance can be significantly adjusted through changing the microstructure parameters.

To identify critical design variables of microstructures, we conducted formula deduction of output voltage and sensitivity. Based on the output voltage formula of the TPS without microstructures [38], the output voltage (*V*) of the TPS with microstructures can be simplified as Equation (1),

(1) $$V=\frac{Qd}{A_{0}\varepsilon_{0}}=\frac{\sigma_{0}Ad}{A_{0}\varepsilon_{0}}$$

where *Q* denotes the induced surface charges on the surface of the microstructures, and *d* is the separation distance, i.e., the compressed distance of the microstructures, and *A*_0_ is the area of the NBR layer, and *A* is the contact area of the microstructures and the NBR layer, and *σ*_0_ is the surface charge density of the microstructures, and *ε*_0_ is the permittivity in vacuum. The sensitivity of the TPS is defined as the ratio of the relative change in the output voltage (*ΔV*) to the change in the pressure (*ΔP*), as provided in Equation (2)

(2) $$\mathrm{Sensitivity}=\frac{\Delta V}{\Delta P}=\frac{\sigma_{0}}{A_{0}\varepsilon_{0}}\frac{\Delta \left(Ad\right)}{\Delta P}$$

**FIGURE 2:**
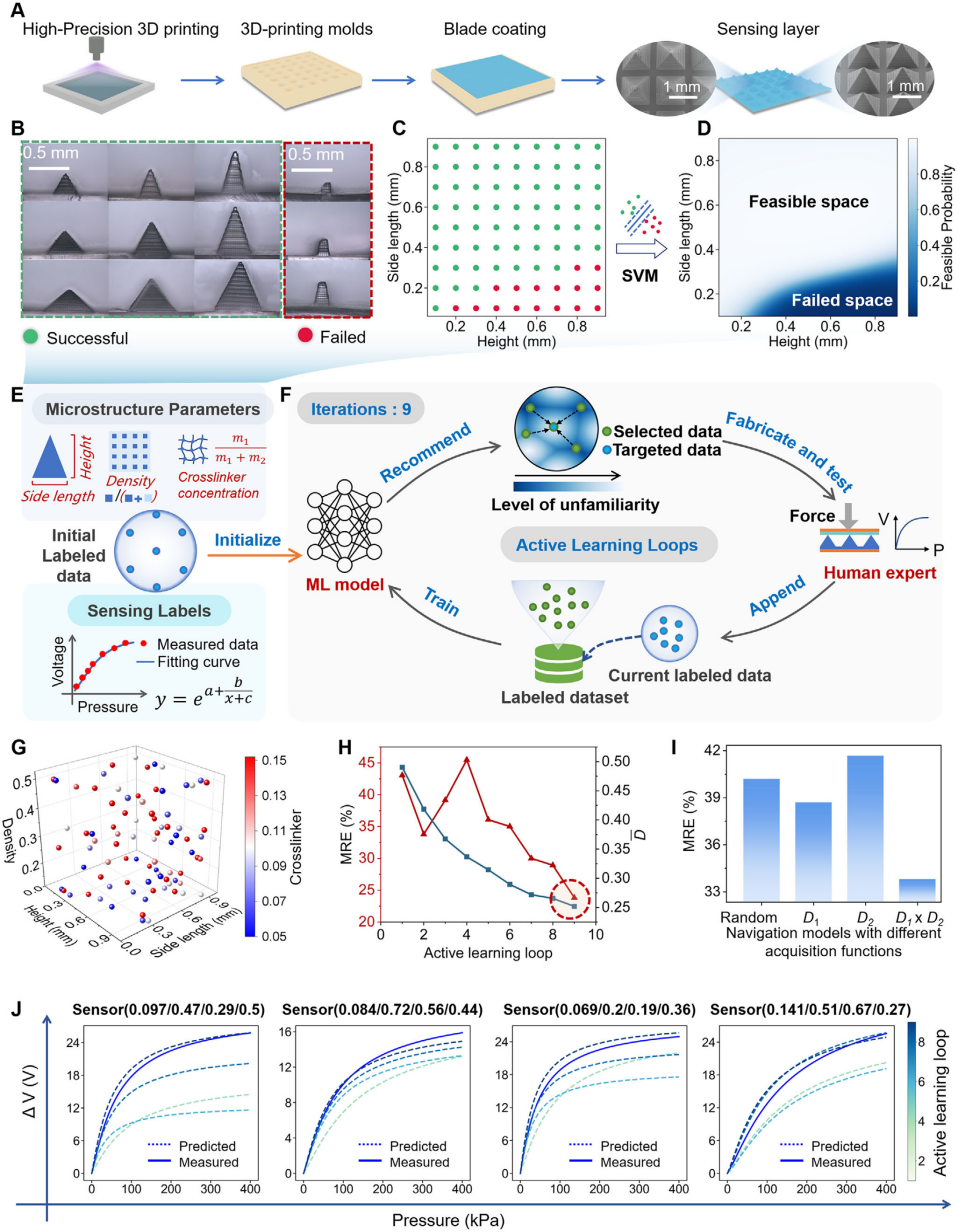
SVM‐based boundary definition and DP‐AL‐enabled effective exploration of the design space. (A) Fabrication process of a PDMS film with quadrangular pyramid microstructures (scale bars: 0.5 mm). (B,C) Comparative optical micrographs showing intact and defective microstructures (scale bars: 0.5 mm) (B), corresponding to successful (green points) and failed (red points) cases in the SVM label space (C). (D) Successful probability heatmap of fabricating the microstructures. (E) Schematic illustration of data features (structure and material) and labels (fitting parameters: *a*, *b*, *c* of pressure‐voltage curves). The *m*_1_ and *m*_2_ denote the mass of the crosslinker and PDMS precursors, respectively. (F) Schematic diagram of the DP‐AL. (G) Selected microstructures distributed in the design space after 9 active learning loops. (H) MRE and $\bar{D}$ of the evolving ANN models during the active learning loops. (I) MRE of the ANN models with different acquisition functions after two active learning loops. The same acquisition function is adopted in the first loop. (J) Comparison of predicted sensing curves (dashed lines) from the ANN models and experimentally measured data (solid lines) across four TPSs. Predicted curves are shown after the 1st, 4th, 7th, and 9th active learning loops, with color gradients indicating the progression of active learning cycles. The microstructure labels are ordered as follows: crosslinker concentration, height (mm), side length (mm), and density.

It can be inferred that the sensitivity can be modulated by tailoring both geometric and material parameters. To systematically engineer the sensing performance of the TPS, we focused on the crosslinker concentration, height, side length, and density of the microstructures. Among them, the crosslinker concentration influences the Young's modulus (*E*) of PDMS positively [39], which is also related to the *σ*_0_ [40]. Meanwhile, the height, side length, density, and *E* collectively determine the dynamic evolution of *A* and *d* under applied pressure, critically affecting not only sensitivity but also linearity and detection range. To verify the impact of these parameters, we fabricated microstructures with varying parameters using molds (Figure S2) that were produced by a high‐precision photocurable 3D printer (Figure 2A,B; Figure S3). The fabricated PDMS films display great flexibility (Figure S4). Following device integration and calibration (Figures S5 and S6), we characterized the pressure‐voltage response curves of TPSs with varied microstructure parameters, which indicate substantial performance variations (Figure S7). Finite element analysis (FEA) further reveals distinct deformation behaviors across microstructures under incremental pressure (Figure S8), confirming the critical role of geometric and material design in tuning sensor performance.

### 2.2 Boundary Definition of Pressure Sensors Design Space

Defining rational boundaries of the design space is essential to ensure sufficient parameter combinations, while avoiding unnecessary attempts. The ranges of the design variables were determined based on expert knowledge and evidence from relevant reported works. A high crosslinker concentration significantly reduces flexibility, whereas a low concentration results in excessive adhesion forces, hindering the separation between triboelectric layers [41]. Consequently, the crosslinker concentration, defined as the mass ratio of crosslinker to the total mass of PDMS precursor and crosslinker, was determined to a range of 0.058–0.16. The height and side length were constrained to a range of 0.1–0.91 mm. The density, defined as the square of the ratio of side length to the sum of side length and spacing (Figure S4), was limited to 0.1–0.5. Based on the fabrication capabilities, each variable is discretized into approximately 20 steps. Therefore, the parameter space encompasses 160 000 possible combinations, making exhaustive experimental evaluation impractical. The observed nonlinear relationships between the design variables and sensing curves further complicates this process (Figures S7 and S8). Therefore, the traditional grid search method is impractical for uncovering the microstructure‐performance relationships accurately.

To build an accurate microstructure‐performance prediction model with limited fabricated sensors, we leveraged a SVM classifier to eliminate infeasible parameters and a DP‐AL model to navigate the design space efficiently. This section focuses on the SVM classifier. During the fabrication of microstructures, the heights and side lengths significantly affect replication fidelity, whereas variations in density and crosslinker concentration have negligible impact. The SVM model was trained to learn the relationship between the microstructures and replication fidelity, enabling excluding poorly conformal parameter regions from the design space. The training data comprises 81 microstructure variants fabricated via 3D‐printed molds, with height and side length varying from 0.1 to 0.9 mm (Figure S9). It should be noted that the workload is reduced by fabricating multiple microstructures on the same molds. Cross‐sectional analysis reveals two distinct morphological classes: intact triangles (green dashed box) and defective trapezoids (red dashed box) (Figure 2B), corresponding to successful (green points) and failed (red points) cases in the SVM label space (Figure 2C). The SVM model achieves a training accuracy of 98.8%. After probability calibration, the SVM model can output a probability value belonging to the successful category for each parameter. Then the calibrated SVM model was utilized to generate a heatmap visualizing the probability of successful preparation across the height‐side length space (Figure 2D). The successful probability >0.8 was selected as the filtering condition. This preprocessing step reduces the design space by 17%, establishing a refined design space for subsequent active learning iterations.

### 2.3 Dual‐Phase Active Learning for Efficient Design Space Exploration

In the active learning section, each training data point integrates microstructural parameters (crosslinker concentration, height, side length, density) and sensing labels (*a*, *b*, *c*) derived from pressure‐voltage curve fitting (Figure 2E). Among various fitting functions, the modified exponential function was identified as the optimal function for modeling the pressure‐voltage curves (Figure S10). Based on the filtered design space, we implemented the DP‐AL algorithm to iteratively selects the most informative data points from the design space, instead of exploring the design space randomly. The flowchart of the DP‐AL algorithm is depicted in Figure 2F. The algorithm operates in two distinct phases, an initial diversity‐focused phase and a subsequent gradient‐guided diversity phase, utilizing different acquisition functions.

In the initial diversity‐focused phase, the acquisition function, called Initial Score, is defined in Equation (3),

(3) $$\mathrm{Initial}\;\mathrm{Score}=D_{1}^{j}$$

where $D_{1}^{j}$ denotes the shortest Euclidian distance between a candidate point (*j*) and all currently selected data points (see Note S3 for details). The first selected point is the center point of the design space. Then, the candidate point with the largest *D*_1_ is recommended by the algorithm, ensuring broad coverage of the design space and establishing an initial labeled pool for the subsequent phase. The schematic illustration of the initial acquisition function is shown in Figure S11A. In the subsequent gradient‐guided diversity phase, the DP‐AL algorithm enters iterative loops of computational sampling and experimentally validation. During each iteration, the DP‐AL algorithm selects data points according to the underlying information embedded in the previous experiments. The selection process is guided by the incremental acquisition function, called Incremental Score, which quantifies the input diversity and output gradients for unlabeled data points. The acquisition function is defined in Equation (4),

(4) $$\mathrm{Incremental}\;\mathrm{Score}=D_{1}^{j}\times D_{2}^{j}$$

where $D_{2}^{j}$ denotes the shortest Euclidian distance between the predicted sensing labels of a candidate data point (*j*) and those of all currently selected data points (see Note S3 for details). This metric ensures that newly selected points simultaneously maximize informational diversity ($D_{1}^{j}$) and performance gradients ($D_{2}^{j}$).

To robustly evaluate the performance gradients, an artificial neural network (ANN) committee is employed, comprising five independently trained models. Each ANN is trained on the current labeled dataset to capture the nonlinear relationships between microstructure features and sensing labels in the data. Predictions are aggregated by averaging the committee outputs, significantly enhancing both the robustness and accuracy of estimation of performance gradients. The DP‐AL algorithm prioritizes data points exhibiting both high diversity and performance gradients. The selected points undergo experimental characterization, after which they are added into the labeled pool. The expanded labeled pool subsequently retrains the ANN committee, closing the iterative loop and progressively refining the model's predictive capability. The Incremental Score effectively balances global exploration of the design space and local exploration of regions where the ANN committee is uncertain, facilitating the selection of high‐information‐density data points. After 9 active learning loops, 90 TPSs were recommended and experimentally validated (10 samples per loop, the initial phase of the DP‐AL is considered as the first loop. See Table S2 for details). Figure 2G shows the distribution of selected microstructures in the design space, while their corresponding pressure‐voltage curves are illustrated in Figure S11B, respectively.

### 2.4 Evaluation of Dual‐Phase Active Learning

The DP‐AL algorithm was evaluated from two perspectives: (1) design space exploration, (2) predictive accuracy evolution, and (3) sampling efficiency benchmarking. First, regarding space exploration, we introduced a uniformity metric, $\bar{D}$, to quantify the homogeneity of design space sampling. $\bar{D}$ is defined as the average shortest Euclidian distance of uniformly distributed data points in the design space and the selected data points (see Note S3 for details). As the number of active learning rounds increases, $\bar{D}$ gradually decreases (Figure 2H), indicating that the DP‐AL algorithm effectively expands the coverage of the design space. Correspondingly, the distribution of selected data points becomes increasingly uniform, as visualized in the design space (Figure S12A–E). The density distribution of all data points further demonstrates the relative uniform distribution (Figure S12F). Notably, local non‐uniform regions in the density map are introduced by the output‐gradient component ($D_{2}^{j}$) of the acquisition function, which prioritizes sampling in performance‐sensitive regions (Note S4 and Figure S13A).

Second, to assess the accuracy of the evolving ANN committee, the mean relative error (MRE) (defined in Note S5) was calculated using a test set of 30 randomly sampled microstructures, which were excluded from the training dataset (see Table S3 for details). As the number of active learning loops increases, the MRE of predicted fit labels decreases from 43.1% to 23.8%, demonstrating improved accuracy (Figure 2H). Additionally, the MREs of predicted voltage, linearity, and sensitivity reach 21%, 2.6%, and 26% across the entire pressure range (0–400 kPa), respectively (Figure S13B). Comparative analyses with other ML models (gradient boosting and decision tree) confirms the superiority of the ANN committee structure in capturing these non‐linear relationships (Figure S13C). The final ANN committee with the lowest MRE was named as the ‘optimal ANN model’.

Third, to validate the sampling efficiency, we conducted comparisons at both experimental and computational levels. In the second cycle of active learning, we experimentally compared different acquisition functions: (1) random selection, (2) *D*_1_‐only, (3) *D*_2_‐only, and (4) *D*_1_  ×  *D*_2_ (Figure 2I). The second loop of the DP‐AL algorithm was executed based on the above acquisition functions, respectively. The ANN committee of *D*_1_  ×  *D*_2_ indicates the smallest MRE, providing initial physical evidence of the effectiveness of balancing input diversity with output gradients. To further quantify the long‐term convergence speed, we utilized the optimal ANN model as a ground truth oracle to simulate the iterative learning process over 9 cycles (detailed in Note S4 and Figure S13D). Benchmarked against established baselines, the DP‐AL strategy demonstrates superior efficiency. Finally, to visually illustrate the refinement process, the prediction curves generated by the ANN model at various stages of active learning loops were compared to the experimentally measured sensing curves in the test set. As shown in Figure 2J, the predicted curves progressively align with the measured curves as the number of active learning loops increases, confirming the effectiveness of the DP‐AL algorithm in refining the ANN model. Additional examples of predicted versus measured curves are provided in Figure S14.

### 2.5 Predictive Sensing Performance of the Multiscale Design Space

**FIGURE 3:**
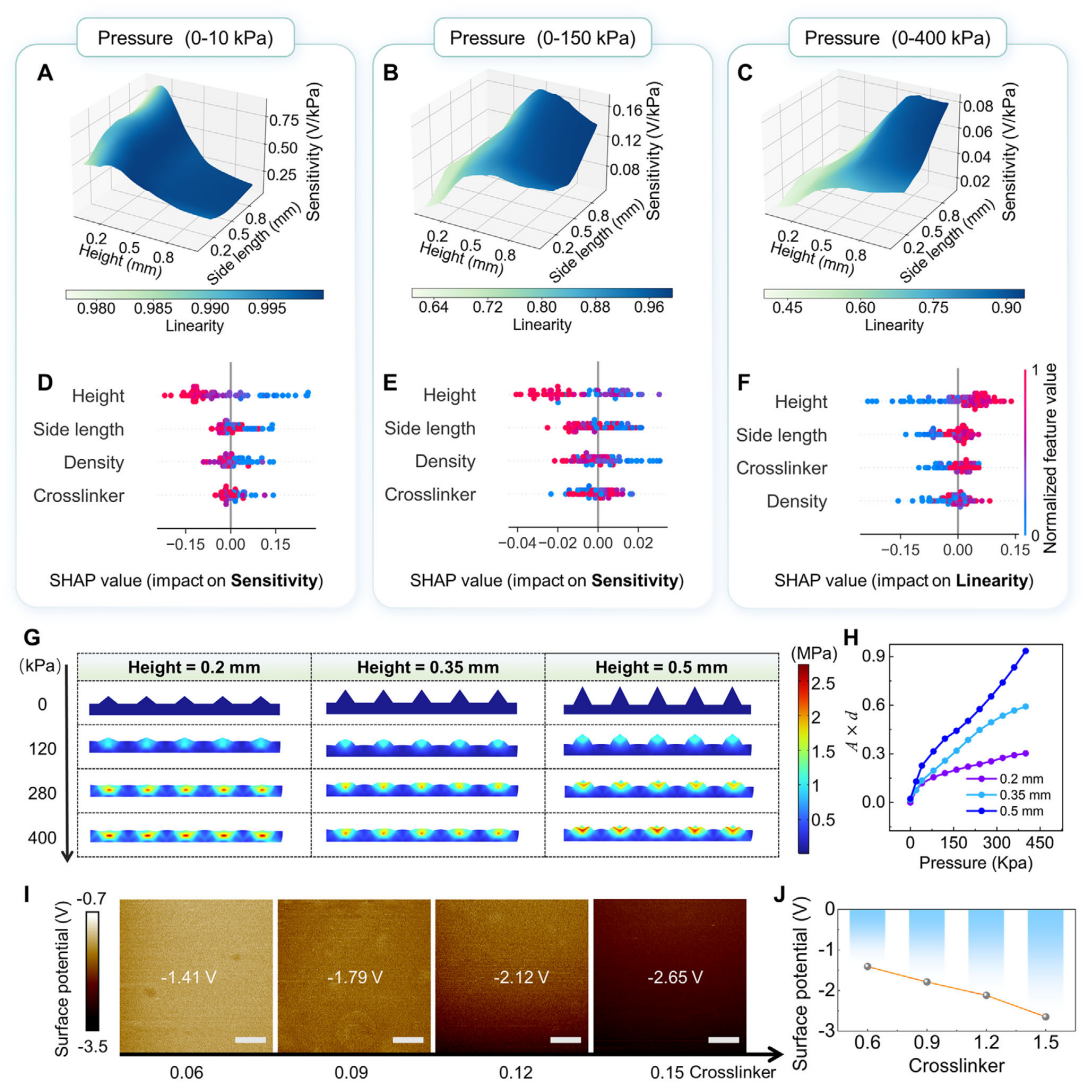
Predictive microstructure‐performance analysis and model interpretation. (A–C) Predictive sensitivity and linearity of pressure ranges: (A) 0–10 kPa, (B) 0–150 kPa, and (C) 0–400 kPa in height‐side length subspace (crosslinker concentration = 0.1, density = 0.1), with linearity represented by color mapping. (D–F) SHAP values (impact on model outputs) of selected microstructures on (D) sensitivity (0–10 kPa), (E) sensitivity (0–150 kPa), and (F) linearity (0–400 kPa). (G) Simulated deformation of microstructures with height of 0.2, 0.35, and 0.5 mm, under increasing pressures (Young's modulus = 2.4 MPa, side length = 0.5 mm, density = 0.25). (H) Simulated relationships between pressure and *A*  ×  *d*. (I,J) KPFM surface potential of PDMS planar films with crosslinker concentrations of 0.6, 0.9, 1.2, and 1.5.

The optimal ANN model serves two primary functions: (1) accurately predicting the sensing performance of microstructural parameters, and (2) enabling the inverse design of microstructures to meet specified sensing performance requirements. This section focuses on analyzing microstructure‐property relationships based on the optimal ANN model. After inputting the microstructure parameters to the optimal ANN model, the model outputs predicted fit labels. Based on the predicted fit labels, the voltages under varying pressure can be calculated (Figure S15). Additionally, leveraging the calculation method of the sensitivity and linearity (Figure S16), the predicted linearity and sensitivity for different pressure ranges can be generated (Figure 3A–C; Figure S17). Among the above figures, the default crosslinker concentration, height, side length, and density were set as 0.11, 0.5 mm, 0.5 mm, and 0.1, respectively. As the pressure range expands from 0–10 kPa to 0–400 kPa, the sensitivity significantly decreases (Figure 3A–C), underscoring the strong coupling between sensitivity and detection range. Moreover, trade‐offs also exist between sensitivity and linearity. For example, in the 0–150 kPa range, the region maximizing sensitivity does not coincide with the region optimizing linearity. These observations highlight inherent conflicts between diverse sensing metrics, which can also be observed in crosslinker‐density subspaces (Figure S17).

### 2.6 Model Interpretation to Discover Microstructure Dominance Mechanism

Although the optimal ANN model exhibits strong predictive capabilities, it remains a black box, necessitating further analysis to elucidate microstructure‐property relationships. Shapley additive explanations (SHAP), a model explanation algorithm, can assess the contribution of individual features to the outputs of any ML model [42]. By applying the modified SHAP analysis (see more details in Note S6), the impact of each microstructural parameter on sensitivity and linearity across targeted pressure ranges can be visualized in the SHAP summary plots (Figure 3D–F). Notably, SHAP values along the horizontal axis quantify the impacts on model outputs. Positive values indicate that a parameter tends to increase the predicted performance metric (e.g., sensitivity or linearity), while negative values suggest the opposite. Parameters are vertically ranked by feature importance, with color gradients representing normalized parameter values.

The height exerts the strongest influence on sensitivity (0–10 kPa), sensitivity (0–150 kPa), and linearity (0–400 kPa). For the density and crosslinker concentration, the importance orders are different for sensitivity and linearity. Furthermore, the influence trends vary significantly across these sensing metrics. For example, the sensitivity (0–10 kPa) is negatively correlated with height, while the linearity (0–400 kPa) indicates the opposite relationship. Conversely, the linearity (0–400 kPa) shows positive correlations with the height, side length, and crosslinker concentration. This indicates that larger and stiffer microstructures (higher height, side length, and crosslinker concentration) are advantageous for maintaining linearity over broader pressure ranges. Furthermore, based on the SHAP analysis, the coupling effect between different parameters on the linearity (0–400 kPa) can be found, with details visualized in the SHAP dependence plots (Figure S18). For example, the most coupling parameter for the height is the side length. It can be deduced that microstructures that are both low and thin, or tall and thick, are advantageous for enhancing the linearity (0–400 kPa) (Figure S18A). By identifying the microstructure‐performance relationships, the SHAP analysis helps bridge the gap between the black‐ box ML model and physical understanding

To demonstrate the results obtained from the SHAP analysis, we conducted FEA and Kelvin probe force microscopy (KPFM) characterization. Based on the sensitivity equation (Equation (2)), it can be inferred that the linearity is related to the ratio of *Δ*(*Ad*) and *ΔP*. The relationships between (*A*  ×  *d*) and pressure (0–400 kPa) are simulated with different heights 0.2, 0.35, and 0.5 mm (Figure 3G). Increasing microstructure height delays deformation saturation, extending the linear response window (Figure 3H), consistent with the SHAP analysis in Figure 3F. Besides, the observed trends in sensitivity (0–10 kPa) and sensitivity (0–150 kPa) concerning crosslinker concentration can be explained by the interplay between two competing factors: the increase of *E* and *σ*_0_ with higher crosslinker concentrations.

KPFM measurements on planar PDMS films with different crosslinker concentrations confirm that the absolute surface potential—a proxy for *σ*_0_—increases with higher crosslinker concentrations (Figure 3I,J). However, higher crosslinker concentrations also lead to an increase in *E*, which generally reduces sensitivity by limiting microstructure deformation (*Δ*(*Ad*)). The relative importance of these two factors varies with the pressure range. Under low‐pressure ranges, microstructures with lower crosslinker concentrations (thus lower *E*) exhibit lager *Δ*(*Ad*). In this condition, *E* dominates the sensitivity response. As the pressure range expands, the *Δ*(*Ad*) of microstructures with different crosslinker concentrations becomes comparable, as the applied pressure is sufficient to overcome the stiffness differences. In this condition, the increased *σ*_0_ at higher crosslinker concentrations become the dominant factor, leading to higher sensitivity. As a result, the sensitivity (0–10 kPa) exhibits a negative relationship with the crosslinker concentration, while the sensitivity (0–150 kPa) possesses a nonlinear relationship. In addition, the competition between *E* and *σ*_0_ also explains to some extent why the crosslinker concentration is the parameter with the least impact.

### 2.7 Inverse Design of Pressure Sensors with Tailored Sensing Performance

**FIGURE 4:**
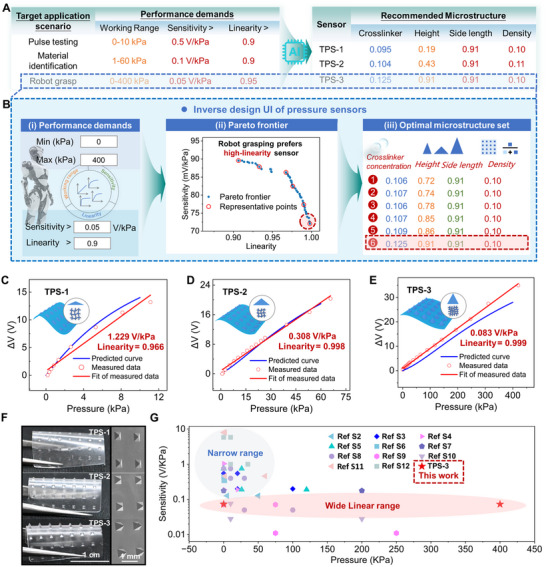
Inverse design of performance‐customizable TPSs for diverse applications. (A) Inverse design processes for various applications. (B) Inverse design UI, consisting of user‐defined sensing performance (i), optimization process and Pareto frontier (ii), and representative microstructures uniformly selected along the Pareto frontier (iii). The red rectangle labels the selected microstructures based on the application preference. (C–E) Predicted, measured, and fit sensing curves for (C) TPS‐1, (D) TPS‐2, and (E) TPS‐3. The values in red indicate the sensitivity and linearity of measured data. (F) Optical and SEM characterization of the three inverse‐designed microstructures. (G) Performance comparison between TPS‐3 with recently published TPSs (thickness < 2 mm).

The performance demands of the TPSs vary significantly across different application scenarios. We proposed an inverse design UI to resolve the dilemma, enabling optimizing the TPSs to target performance. The workflow of the UI is illustrated in Figure 4A,B, based on the optimal ANN model and a multi‐objective optimization algorithm (see Figure S19 for details). The Non‐Dominated Sorting Genetic Algorithm II (NSGA‐II) was employed in this work as the core optimization engine to resolve the trade‐offs among performance metrics of the TPS. This choice is motivated by NSGA‐II's ability to handle large design spaces with multiple constraints. The inverse design problem is formulated as a constrained multi‐objective optimization that simultaneously maximizes sensitivity and linearity within user‐specified detection ranges, defined as Equation (5),

(5) $$\left[\begin{array}{c} \underset{x}{\max\;}\left\{\mathrm{Linearity}\left(x\right),\mathrm{Sensitivity}\left(x\right)\right\}\\ s.\;t.\;p_{\min}\leq \mathrm{Detection}\;\mathrm{Range}\left(x\right)\leq p_{\max},\\ \begin{array}{c} \mathrm{Linearity}\left(x\right)\geq L_{\min},\\ \mathrm{Sensitivity}\left(x\right)\;\geq \;S_{\min},\;\mathrm{SVM}\left(x\right)\;\geq 0.8 \end{array} \end{array}\right]$$

where *P*_min_ and *P*_max_ denote the lower and upper limits of the detection range, *L*_min_ and *S*_min_ denote the lower limits of linearity and sensitivity, SVM denotes the trained SVM model, *x* denotes the microstructure parameter in the design space. Through inputting the required detection range into the UI, the UI can rapidly output Pareto frontiers of linearity and sensitivity. The Pareto frontier represents the set of optimal trade‐off solutions where no objective can be improved without worsening at least one other objective. The UI allows the discovery of microstructures with optimal trade‐offs in the linearity, sensitivity, and detection range, which enables the customizable design of the TPSs. The customizable design lies in a strategic performance reallocation to tunnels the coupling of the multiple metrics, i.e., deliberately sacrificing non‐critical metrics to maximize mission‐essential metrics.

To validate the customizable capability, the UI was employed to design TPSs for diverse applications: pulse monitoring, material identification, and robot grasping. After inputting the performance requirements in Figure 4A, the optimal microstructures are recommended by the UI. Taking the design of the robot grasping sensor as an example to illustrate the workflow of the inverse design UI. Based on the performance requirements (Figure 4B(i)), the optimization process is driven by NSGA‐II. During each iteration, linearity and sensitivity are calculated from the predicted pressure‐voltage curves, while the SVM classifier assesses manufacturability. These evaluations guide NSGA‐II to generate Pareto frontiers that capture the trade‐offs between linearity and sensitivity (Figure 4B(ii)). Six representative optimal microstructures are selected uniformly along the Pareto frontier (Figure 4B(iii)). Considering the robot grasping sensor prioritizes linearity, the optimal solution with the highest linearity was selected to be fabricated by the 3D printing machine.

Additionally, the material identification sensor prioritizes the balance between linearity and sensitivity, while the pulse sensor focuses on higher sensitivity. The corresponding parameters selected from the Pareto frontier were experimentally fabricated (Figure S20). The actual test values closely match the predicted values (Figure 4C–E), confirming the accuracy and reliability of the inverse design UI. The inverse‐designed TPSs exhibit high sensitivity (1.229 V/kPa), high linearity (*R* ^2^ = 0.999), and wide detection range (0–400 kPa), covering the design demands derived from the application scenarios. Moreover, while our primary optimization targets are sensitivity and linearity, the detection range is implicitly maximized by dynamically adjusting the constraints, enabling three‐objective optimization that breaks the coupling of multiple metrics. Besides, the frequency response (1–4 Hz) test and durability test (8000 cycles) demonstrate superb stability of the inverse‐designed sensor (Figure S21). In addition, we compared NSGA‐II with the Bayesian optimization (BO) algorithm in solving the multi‐objective optimization problem. While the BO algorithm requires iterative weight adjustments to generate single solutions (Figure S22), NSGA‐II directly maps the complete Pareto frontier with superior computational efficiency. The inverse design processes for TPS‐1, TPS‐2, and TPS‐3 are visualized in Movies S1–S3, based on the inverse design UI, demonstrating efficient and customizable design ability.

Optical and SEM characterization reveal three distinct, high‐precision, inverse‐designed microstructures (Figure 4F). The inverse‐designed TPSs exhibit high sensitivity and linearity across wide detection range (Figure 4G; Table S1). Notably, compared with previously reported TPSs, the TPS‐3 first achieves a continuous linear response across 0–400 kPa, eliminating nonlinear transition zones. These results highlight that our inverse design strategy not only decouples the coupling of multiple performance metrics but also exhibits strong customizability for diverse application requirements (i.e., optimizing key performance indicators at the expense of lower‐priority metrics).

### 2.8 Generality Demonstration of Inverse Design to Different Materials, Structures, and Applications

**FIGURE 5:**
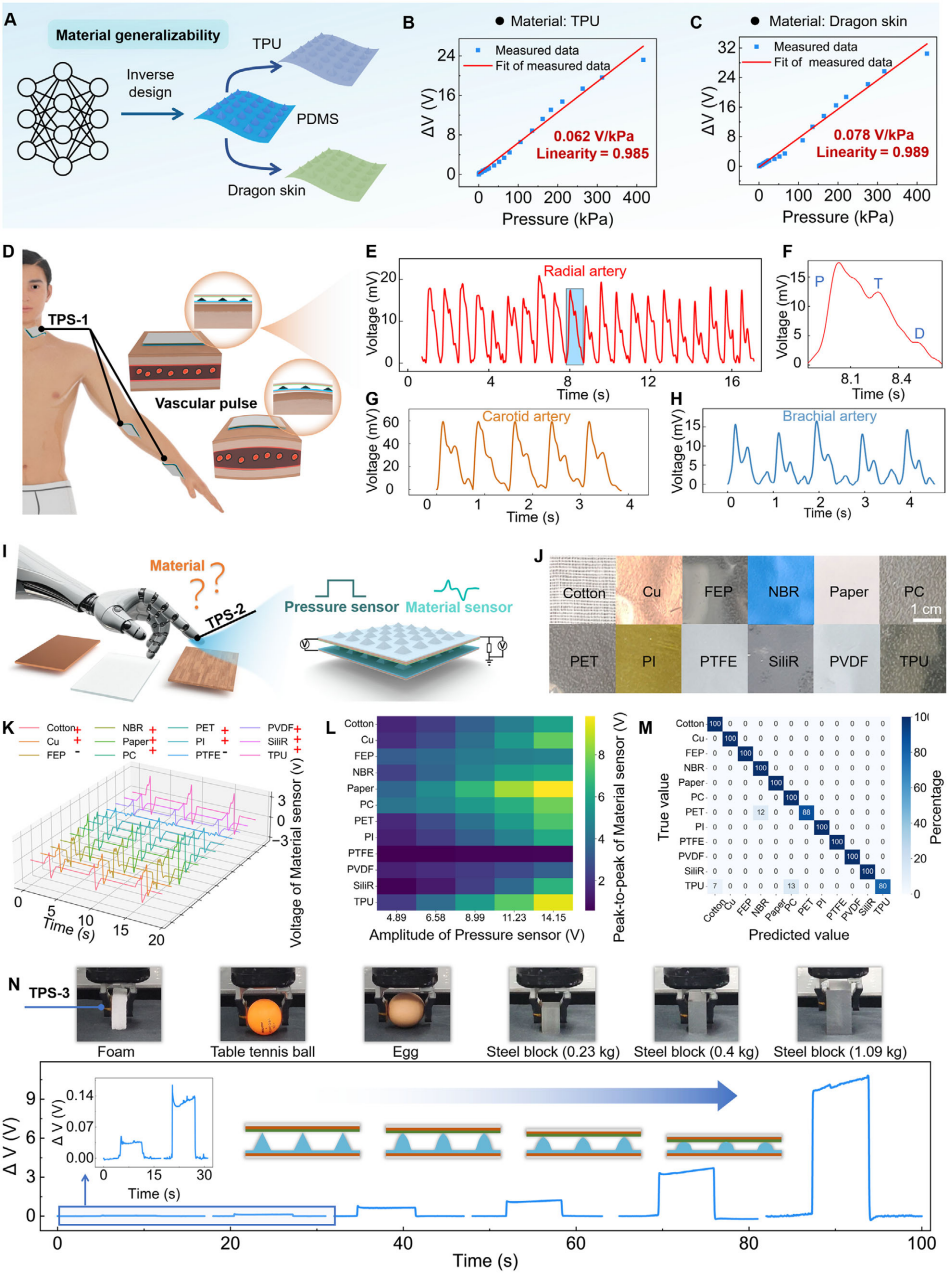
Generalizability demonstration of inverse design to different materials and applications. (A) Schematic diagram of material generalizability. (B,C) Measured and fitting curves for (B) TPU‐TPS and (C) Dragonskin‐TPS. (D) Schematic illustration of pulse monitoring. (E–H) Radial artery (E), enlarged Radial artery (F), Carotid artery (G), and Brachial artery (H) pulse signals measured from a healthy volunteer. (I) Schematic illustration of a robot hand integrated with the material sensing module that comprises of a pressure sensor (TPS‐2) and a material sensor. (J) Photograph of materials to be identified. (K) Voltage waveforms of the material sensor for 12 materials under the same pressure. ‘+’ indicates peak‐then‐valley responses, while ‘−’ indicates valley‐then‐peak responses. (L) Peak‐to‐peak voltage values along with the pressure sensor's output for 12 materials. (M) Confusion matrix for material identification. (N) Voltage signals of the pressure sensor when the robotic gripping jaw grasps the foam (1.1 g), table tennis (2.7 g), egg (0.05 kg), steel block (0.23 kg), steel block (0.4 kg), and steel block (1.09 kg). The inset shows the enlarged voltage curves of foam and table tennis.

The inverse design methodology demonstrates generality of its structural optimization strategy to other elastic materials. By implementing a modulus mapping strategy (detailed in Note S7 and Figure S23), the software can be readily extended to materials beyond PDMS without requiring additional training (Figure 5A). To validate this, we input the performance targets of TPS‐3 along with the respective modulus constraints of new materials (thermoplastic polyurethane (TPU)) and Dragonskin) into the software. Based on these inputs, the software recommended the same optimized parameter set (0.91/0.91/0.1). The parameter set was subsequently used to fabricate microstructure layers. This microstructure layer was then physically assembled with its corresponding planar triboelectric layer (PDMS for TPU‐TPS, and NBR for Dragonskin‐TPS) to form the functional pressure sensor device. They exhibit good linearity and sensitivity, *R* ^2^ = 0.985, 0.062 V/kPa (TPU‐TPS), *R* ^2^ = 0.989, 0.078 V/kPa (Dragonskin‐TPS) (Figure 5B,C). This proves the generality of our inverse design approach in structural optimization. Moreover, the inverse design UI, initially developed for dual‐electrode TPSs, is also applicable to single‐electrode configurations. The adaptation is due to their shared dependence on microstructure‐mediated pressure‐response characteristics [43]. The single‐electrode TPS adopts the same inverse‐designed microstructures of TPS‐2. The pressure‐voltage curves for four materials (Cu, Paper, PI, and TPU) demonstrate excellent linearity (Figure S24A), confirming the UI's generality to design single‐electrode TENG sensors.

Application validation of the TPSs is shown in Figure 5D–N. In the pulse test, the pulse sensor was tested on a healthy volunteer's carotid, brachial, and radial arteries (Figure 5D). Continuous measurements of the wrist arterial pulse show stable waveforms (Figure 5E), with a magnified view revealing three characteristic peaks (Figure 5F). The sensor also exhibits high sensitivity in detecting pulses from the carotid and brachial arteries (Figure 5G,H), validating its potential for healthcare monitoring. As shown in Figure 5I, accurate material identification of 12 materials (Figure 5J) can be achieved by extracting features from the sensing module's signals using an ANN model. The module consists of a dual‐electrode TPS and a single‐electrode triboelectric material sensor, which are vertically stacked and adopt the same microstructures. While the material sensor generates distinct voltage waveforms for different materials, its output is inherently coupled with contact pressure. Enabling precise material identification thus requires accurate decoupling of the pressure dependency from the material‐specific signal. The inverse‐designed microstructures are crucial because they ensure both the pressure sensor and the material sensor exhibit a linear response to pressure. This dual‐linearity ensures that the voltage outputs of both sensors scale predictably with pressure, providing linear data inputs that significantly simplifies the training of the ANN model.

The pressure sensor compensates for this pressure dependency, enabling precise material identification. The inverse‐designed microstructures for both sensors ensure a linear response to pressure, simplifying the training of the ANN model.

To train the ANN model, the outputs of the pressure and material sensors were tested 10 times for 12 different materials at 15, 20, 25, 35, and 45 kPa, yielding 600 data points (80% for training, 20% for testing). The voltage waveforms for the 12 materials at 25 kPa are shown in Figure 5K, exhibiting either peak‐then‐valley (e.g., cotton, NBR, denoted as ‘+’) or valley‐then‐peak (e.g., PTFE, FEP, denoted as ‘−’) responses. The peak, valley, and peak‐to‐peak voltage values, along with the pressure sensor's output, create unique “fingerprints” for each material (Figure S24B,C; Figure 5L). The trained ANN model achieves 96.7% accuracy on the test set, with 100% accuracy for all materials except PET and TPU (Figure 5M). This high accuracy achieved under varying pressures directly validates the effectiveness of the inverse‐designed microstructures in generating the necessary linear data required for material identification. In robot grasping application, the pressure sensor was mounted on the gripper of a robotic arm to grasp objects of varying weights: foam (1.1 g), a ping‐pong ball (2.7 g), an egg (0.05 kg), a small iron block (0.23 kg), a medium iron block (0.4 kg), and a large iron block (1.09 kg). The delta voltages increase with grasping weight (Figure 5N), demonstrating the sensor's linearity and highlighting its potential for robotic control applications. These application demonstrations can be seen in Movies S1–S3.

## 3 Discussion

In this work, we have developed a multi‐objective inverse design methodology that achieves rapid on‐demand customization of TPSs. By integrating SVM‐based pre‐screening and DP‐AL‐guided modeling, the methodology establishes a prediction model based on sparse experimental data (from 90 sensors), forecasting full pressure‐voltage response across 0–400 kPa accurately. Assisted by a multi‐objective optimization algorithm, the prediction model empowers an inverse design software, facilitating the discovery of Pareto‐optimal microstructures for customized linearity, sensitivity, and detection range within seconds. The inverse‐designed sensors exhibit outstanding sensing performance: 96.7% accuracy in material identification (12 materials), pulse monitoring (1.2 V/kPa), and robotic grasping (*R* ^2^ = 0.999, 0–400 kPa). Notably, in contrast to previously reported TPSs, our customized TPS first demonstrates a continuous linear response across the entire 0–400 kPa range, eliminating traditional nonlinear transition zones. Additionally, the generalizability of the inverse design was demonstrated across diverse materials (TPU, Dragonskin). The interpretable machine learning algorithm reveals design mechanisms for microstructure optimization, validated by the FEA and KPFM characterization. The comprehensive evaluation of design efficiency demonstrates the superiority of our inverse design approach over relevant forward and inverse design methodologies (Figure 1D; Note S1 and Table S1).

While this work evidences the transformative potential of ML‐driven inverse design for TPSs, several avenues for future research remain. First, leveraging the inverse design software to deal with more challenging sensing requirements (e.g., linear range > 1000 kPa) to validate the extreme design ability. Second, the methodology could be extended to incorporate additional performance metrics, such as durability and response time, to enhance the versatility of TPSs. Third, investigating high‐throughput methods of fabricating and calibrating pressure sensors to accelerate the data acquisition efficiency. By addressing these challenges, the proposed methodology has the potential to revolutionize the design approaches of flexible sensors.

## 4 Methods

### 4.1 Fabrication of PDMS, TPU, and Dragonskin with Microstructures

PDMS: The template (Figure S2) for patterning microstructures was fabricated by a high‐precision photocurable 3D printing machine with a resolution: 30 µm in XY plane, 5 µm in Z axis (Octavelight R1‐30 µm, Dongguan Baduguang Technology Co., Ltd, China). PDMS precursors and curing agents were purchased from Dow corning (sylgard 184, USA). The fabrication process of the PDMS films with microstructures is shown in Figure 2a. First, the PDMS precursor and crosslinker were mixed in the specified ratio and poured into the microstructure mold. After degassing to remove bubbles, an excess PDMS solution on the template surface was scraped off. Finally, the solution was cured at 100°C for 2 h, after which a microstructure PDMS film can be peeled off.

**TPU**: TPU granules (Shore 35A, Shenzhen Guangyuan Plastic Chemical Co., Ltd., China) were weighed and dissolved in dimethylacetamide (Shanghai Macklin Biochemical Technology Co., Ltd., China) under magnetic stirring (600 rpm) at 50°C for 6 h. Then TPU solution was under the same fabricating process of the microstructure PDMS, after which a TPU film can be peeled off.
**Dragonskin**: Part A (base elastomer) and Part B (curing agent) of Dragonskin 30 (Smooth‐On, Inc., USA) were mixed at a 1:1 weight ratio. After processed with the same fabricating process of the microstructure PDMS, a Dragonskin film can be peeled off.

### 4.2 Fabrication of the TPS

The microstructures with varying crosslinker concentration, height, side length, and density can be obtained by the 3D printing‐enabled technique (Figures S3 and S9). Then, NBR films (Ammex, USA) and patterned PDMS films were cut by a die cutting machine. PI‐Cu films were customized flexible printed circuit boards (Shenzhen Ruixing Express PCB Co., Ltd, China). Next, a TPS is fabricated by sequentially integrating two PI‐Cu films (thickness: 43 µm), NBR films (thickness: 50 µm), and patterned PDMS films in a layered structure (Figure S5). The size of Cu, NBR films, and patterned PDMS films is 2 cm × 2 cm. The TPU‐TPS and Dragonskin‐TPS follow the same fabrication process as the PDMS‐TPS. The planar triboelectric layer is PDMS for the TPU‐TPS, while the planar layer is still NBR for the Dragonskin‐TPS.

### 4.3 Characterizations

Surface morphology of microstructures in Figure 2A was characterized by a scanning electron microscope (SEM) (SU3500, Hitachi, Japan). Side view of microstructures was characterized by an optical microscope (MCK‐6RC, Caikon, China). The surface potential of PDMS plantar films with different crosslinker concentrations was characterized by a Kelvin probe force microscopy (KPFM) (NanoIR2‐FS, Bruker, USA). The force calibration system of the TPS is shown in Figure S6A. The upper electrode and NBR film are fixed to the Instron indenter, while the lower electrode and PDMS film are fixed to a compression testing machine (34‐0C, Instron, USA). In the initial position, the tip of the microstructure was in contact with the NBR film. To characterize the pressure‐sensing performance of the TPS, dynamic pressure ranging from 0 to 400 kPa were applied using the Instron machine. The open circuit voltage was collected by an electrometer (Keithley 6514, Tektronix, USA), transmitted to a PC by a data acquisition card (USB‐6001, National Instruments, USA), and processed by a LabVIEW program. Pressure‐voltage curves were obtained by peak analysis of the pressure and voltage curves (Figure S6B–D). For the data collection in material identification, the material sensor was connected in series with a 250 MΩ resistor, the voltage of which was directly measured by the data acquisition card.

### 4.4 ML Models and Optimization Algorithm in the Inverse Design Methodology

**SVM classifier**: The SVM classifier employed to filter poorly conformal parameter combinations was implemented in Python with a scikit‐learn package. We selected a radial basis function (RBF) as the kernel function of the SVM classifier to deal with the non‐linear data points. The reasons for training the SVM classifier on the entire dataset, rather than splitting it into training and testing subsets, are as follows: The SVM classifier serving as a data‐driven filter, instead of a prediction model. Moreover, the SVM classifier is based on identifying the maximum margin hyperplane, which demonstrates robustness against noise and overfitting in low‐dimensional spaces (e.g., when using only height and side length as features).
**ANN model in DP‐AL algorithm**: An ensemble committee of five multilayer perceptron (MLP) networks (4 × 15 × 17 × 15 × 3) was trained on the labeled dataset to model the underlying relationships between microstructural features and pressure‐voltage sensing curves. Each MLP consists of an input layer (four features: crosslinker concentration, height, side length, and density), followed by hidden layers with ReLU activation, and a final layer. The final linear output layer produces three fit parameters. The models were optimized using the Adam algorithm, and the committee's prediction was obtained by averaging the outputs of all five MLPs.
**ANN model in material identification**: The ANN model adopts the MLP network with an architecture of 4 × 20 × 22 × 22 × 20 × 12. The input layer consists of four features: first extremum (peak or valley), second extremum (peak or valley), and peak‐to‐peak voltage values of material sensor waveforms, along with the pressure sensor's output. The final output layer generates the material type. Each layer of the MLP, except for the final layer, was followed by ReLU activation function and was trained using Adam optimization.
**NSGA‐II algorithm**: NSGA‐II, implemented in Python with a pymoo package, was configured with a population size of 50 and a maximum of 50 generations. During each generation, the fitness of individuals is evaluated using the optimal ANN model to predict performance metrics. Linearity and sensitivity are calculated from the predicted pressure‐voltage curves, while the SVM classifier assesses manufacturability. These evaluations guide NSGA‐II in performing non‐dominated sorting and crowding distance calculations, generating Pareto frontier that captures the trade‐offs between linearity and sensitivity. The aforementioned models were implemented in Python 3.11.5 using the PyTorch methodology (version 2.4.1).

### 4.5 Finite Element Analysis

Considering PDMS is a hyper‐elastic material, we selected Neo‐Hookean model to simulate the mechanical property of PDMS with microstructures based on COMSOL simulation software. The density and Poisson's ratio were set as 970 kg/m^3^ and 0.49, respectively.

## Author Contributions

B.C.W., D.P.K., and G.Y. conceived the idea and designed the research. B.C.W. and D.P.K. designed the ML models and algorithms. B.C.W., Z.A.H., and J.K.L. carried out the FEA, device fabrication, and characterizations. Z.K.D. and H.H.L. carried out the data collection for material identification. M.K.W. carried out the manipulation of robotic arm in applications. Y.Y.L., M.J.D., Z.Q.Y., H.H.L., K.C.X., G.Y., H.Y.Y, and J.C. helped in the process of writing, review and editing.

## Conflicts of Interest

The authors declare no conflicts of interest.

## Supporting information




**Supporting File 1**: advs74088‐sup‐0001‐SuppMat.pdf.

**Supporting File 2**: advs74088‐sup‐0002‐MovieS1.mp4.

**Supporting File 3**: advs74088‐sup‐0003‐MovieS2.mp4.

**Supporting File 4**: advs74088‐sup‐0004‐MovieS3.mp4.

## Data Availability

The data that support the findings of this study are available from the corresponding author upon reasonable request. The Python code to implement the core ML tasks within this study are available from GitHub (https://github.com/baocheng12AIZJU/inverse_design_of_pressure_sensor).

## References

[1] A. Zimmerman , L. Bai , and D. D. Ginty , “The Gentle Touch Receptors of Mammalian Skin,” Science 346 (2014): 950–954. 25414303 10.1126/science.1254229 PMC4450345

[2] N. Bai , Y. Xue , S. Chen , et al., “A Robotic Sensory System with High Spatiotemporal Resolution for Texture Recognition,” Nature Communications 14 (2023): 7121. 10.1038/s41467-023-42722-4 PMC10645869 37963866

[3] D. Kong , G. Yang , G. Pang , et al., “Bioinspired Co‐Design of Tactile Sensor and Deep Learning Algorithm for Human‐Robot Interaction,” Advanced Intelligent Systems 4 (2022): 2200050.

[4] W. Lin , Y. Xu , S. Yu , et al., “Highly Programmable Haptic Decoding and Self‐Adaptive Spatiotemporal Feedback toward Embodied Intelligence,” Advanced Functional Materials 35 (2025): 2500633.

[5] Y. Fang , Y. Zou , J. Xu , et al., “Ambulatory Cardiovascular Monitoring via a Machine‐Learning‐Assisted Textile Triboelectric Sensor,” Advanced Materials 33 (2021): 2104178. 10.1002/adma.202104178 PMC9205313 34467585

[6] W. Fan , Q. He , K. Meng , et al., “Machine‐knitted Washable Sensor Array Textile for Precise Epidermal Physiological Signal Monitoring,” Science Advances 6 (2020): aay2840. 10.1126/sciadv.aay2840 PMC7069695 32201720

[7] S. W. Kim , J. Lee , H. J. Ko , et al., “Mechanically Robust and Linearly Sensitive Soft Piezoresistive Pressure Sensor for a Wearable Human–Robot Interaction System,” ACS Nano 18 (2024): 3151–3160. 38235650 10.1021/acsnano.3c09016

[8] B. Shao , M. Lu , T. Wu , et al., “Large‐area, Untethered, Metamorphic, and Omnidirectionally Stretchable Multiplexing Self‐powered Triboelectric Skins,” Nature Communications 15 (2024): 1238. 10.1038/s41467-024-45611-6 PMC10858173 38336848

[9] C. Wei , W. Lin , L. Wang , et al., “Conformal Human–Machine Integration Using Highly Bending‐Insensitive, Unpixelated, and Waterproof Epidermal Electronics toward Metaverse,” Nano‐Micro Letters 15 (2023): 199. 37582974 10.1007/s40820-023-01176-5 PMC10427580

[10] Z. Huang , S. Yu , Y. Xu , et al., “In‐Sensor Tactile Fusion and Logic for Accurate Intention Recognition,” Advanced Materials 36 (2024): 2407329. 10.1002/adma.202407329 38966893

[11] J. Suo , Y. Liu , J. Wang , et al., “AI‐Enabled Soft Sensing Array for Simultaneous Detection of Muscle Deformation and Mechanomyography for Metaverse Somatosensory Interaction,” Advanced Science 11 (2024): 2305025. 38376001 10.1002/advs.202305025 PMC11040359

[12] Y. Zhou , X. Xiao , G. Chen , X. Zhao , and J. Chen , “Self‐powered Sensing Technologies for human Metaverse Interfacing,” Joule 6 (2022): 1381–1389.

[13] J. Liu , Z. Wen , H. Lei , Z. Gao , and X. Sun , “A Liquid–Solid Interface‐Based Triboelectric Tactile Sensor with Ultrahigh Sensitivity of 21.48 kPa^−1^ ,” Nano‐Micro Letters 14 (2022): 88. 35362790 10.1007/s40820-022-00831-7 PMC8975924

[14] S. Qin , P. Yang , Z. Liu , et al., “Triboelectric Sensor with Ultra‐wide Linear Range Based on Water‐containing Elastomer and Ion‐rich Interface,” Nature Communications 15 (2024): 10640. 10.1038/s41467-024-54980-x PMC11624205 39643620

[15] X. Shi , X. Fan , Y. Zhu , et al., “Pushing Detectability and Sensitivity for Subtle Force to New Limits with Shrinkable Nanochannel Structured Aerogel,” Nature Communications 13 (2022): 1119. 10.1038/s41467-022-28760-4 PMC8891261 35236851

[16] M. Gu , B. Zhao , J. Gao , et al., “Nested‐Cell Architecture and Molecular Surface Modification Enabled 10 Megapascals Range High Sensitivity Flexible Pressure Sensors for Application in Extreme Environment,” Advanced Functional Materials 34 (2024): 2400494.

[17] W. Xiong , F. Zhang , S. Qu , L. Yin , K. Li , and Y. Huang , “Marangoni‐driven Deterministic Formation of Softer, Hollow Microstructures for Sensitivity‐enhanced Tactile System,” Nature Communications 15 (2024): 5596. 10.1038/s41467-024-49864-z PMC11222500 38961075

[18] W. Cheng , X. Wang , Z. Xiong , et al., “Frictionless Multiphasic Interface for near‐ideal Aero‐elastic Pressure Sensing,” Nature Materials 22 (2023): 1352–1360. 37592030 10.1038/s41563-023-01628-8

[19] X. Zhao , Y. Zhou , A. Li , et al., “A Self‐filtering Liquid Acoustic Sensor for Voice Recognition,” Nature Electronics 7 (2024): 924–932. 10.1038/s41928-024-01196-y PMC13095302 42016470

[20] X. Zhao , Y. Zhou , Y. Song , et al., “Permanent Fluidic Magnets for Liquid Bioelectronics,” Nature Materials 23 (2024): 703–710. 38671161 10.1038/s41563-024-01802-6 PMC13078906

[21] H. Zhou , Y. Gui , G. Gu , et al., “A Plantar Pressure Detection and Gait Analysis System Based on Flexible Triboelectric Pressure Sensor Array and Deep Learning,” Small 21 (2024): 2405064. 10.1002/smll.202405064 39473332

[22] S. R. A. Ruth , V. R. Feig , H. Tran , and Z. Bao , “Microengineering Pressure Sensor Active Layers for Improved Performance,” Advanced Functional Materials 30 (2020): 2003491.

[23] Y. Jin , S. Xue , and Y. He , “Flexible Pressure Sensors Enhanced by 3D‐Printed Microstructures,” Advanced Materials 37 (2025): 2500076. 10.1002/adma.202500076 40249136

[24] Y. Luo , M. R. Abidian , J. Ahn , et al., “Technology Roadmap for Flexible Sensors,” ACS Nano 17 (2023): 5211–5295. 36892156 10.1021/acsnano.2c12606 PMC11223676

[25] X. Zheng , X. Zhang , T. T. Chen , and I. Watanabe , “Deep Learning in Mechanical Metamaterials: From Prediction and Generation to Inverse Design,” Advanced Materials 35 (2023): 2302530. 10.1002/adma.202302530 37332101

[26] J. Bastek and D. M. Kochmann , “Inverse Design of Nonlinear Mechanical Metamaterials via Video Denoising Diffusion Models,” Nature Machine Intelligence 5 (2023): 1466–1475.

[27] S. Molesky , Z. Lin , A. Y. Piggott , W. Jin , J. Vucković , and A. W. Rodriguez , “Inverse Design in Nanophotonics,” Nature Photonics 12 (2018): 659–670.

[28] H. Yang , J. Li , K. Z. Lim , et al., “Automatic Strain Sensor Design via Active Learning and Data Augmentation for Soft Machines,” Nature Machine Intelligence 4 (2022): 84–94.

[29] C. S. Ha , D. Yao , Z. Xu , et al., “Rapid Inverse Design of Metamaterials Based on Prescribed Mechanical Behavior through Machine Learning,” Nature Communications 14 (2023): 5765. 10.1038/s41467-023-40854-1 PMC10505607 37718343

[30] B. Li , B. Deng , W. Shou , et al., “Computational Discovery of Microstructured Composites with Optimal Stiffness‐toughness Trade‐offs,” Science Advances 10 (2024): adk4284. 10.1126/sciadv.adk4284 PMC10836719 38306429

[31] S. Ekins , A. C. Puhl , K. M. Zorn , et al., “Exploiting Machine Learning for End‐to‐end Drug Discovery and Development,” Nature Materials 18 (2019): 435–441. 31000803 10.1038/s41563-019-0338-z PMC6594828

[32] J. Moon , W. Beker , M. Siek , et al., “Active Learning Guides Discovery of a Champion Four‐metal Perovskite Oxide for Oxygen Evolution Electrocatalysis,” Nature Materials 23 (2023): 108–115. 37919351 10.1038/s41563-023-01707-w

[33] C. Xu , S. A. Solomon , and W. Gao , “Artificial Intelligence‐powered Electronic Skin,” Nature Machine Intelligence 5 (2023): 1344–1355. 10.1038/s42256-023-00760-z PMC10868719 38370145

[34] Z. Ballard , C. Brown , A. M. Madni , and A. Ozcan , “Machine Learning and Computation‐enabled Intelligent Sensor Design,” Nature Machine Intelligence 3 (2021): 556–565.

[35] Z. Liu , M. Cai , S. Hong , et al., “Data‐driven Inverse Design of Flexible Pressure Sensors,” Proceedings of the National Academy of Sciences 121 (2024): 2320222121. 10.1073/pnas.2320222121 PMC11252744 38954542

[36] H. Zou , Y. Zhang , L. Guo , et al., “Quantifying the Triboelectric Series,” Nature Communications 10 (2019): 1427. 10.1038/s41467-019-09461-x PMC6441076 30926850

[37] Z. Shi , L. Meng , X. Shi , et al., “Morphological Engineering of Sensing Materials for Flexible Pressure Sensors and Artificial Intelligence Applications,” Nano‐Micro Letters 14 (2022): 141. 35789444 10.1007/s40820-022-00874-w PMC9256895

[38] S. Niu , S. Wang , L. Lin , et al., “Theoretical Study of Contact‐mode Triboelectric Nanogenerators as an Effective Power Source,” Energy & Environmental Science 6 (2013): 3576.

[39] R. Seghir and S. Arscott , “Extended PDMS Stiffness Range for Flexible Systems,” Sensors and Actuators A: Physical 230 (2015): 33–39.

[40] Z. Sun , W. Yang , P. Chen , Y. Zhang , X. Wang , and Y. Hu , “Effects of PDMS Base/Agent Ratios and Texture Sizes on the Electrical Performance of Triboelectric Nanogenerators,” Advanced Materials Interfaces 9 (2022): 2102139.

[41] J. Chen , K. E. Wright , and M. A. Birch , “Nanoscale Viscoelastic Properties and Adhesion of Polydimethylsiloxane for Tissue Engineering,” Acta Mechanica Sinica 30 (2014): 2–6.

[42] S. M. Lundberg and S. Lee , “A Unified Approach to Interpreting Model Predictions,” Advances in neural information processing systems 30 (2017): 4765–4774.

[43] S. Niu , Y. Liu , S. Wang , et al., “Theoretical Investigation and Structural Optimization of Single‐Electrode Triboelectric Nanogenerators,” Advanced Functional Materials 24 (2014): 3332–3340.

