# Supplementary material for: Customizing Tactile Sensors via Machine Learning‐Driven Inverse Design
Source: Adv Sci (Weinh). 2026 Jan 28;13(29):e24250. doi: 10.1002/advs.202524250 (PMC13205881; doi:10.1002/advs.202524250)
Supplement: Supplementary file 1 — Supporting File 1: advs74088‐sup‐0001‐SuppMat.pdf. [file ADVS-13-e24250-s003.pdf]

Supplementary Materials for

Customizing Tactile Sensors via Machine Learning-driven Inverse Design

Baocheng Wang[1], Depeng Kong[1,2], Zhiao He[1], Jikai Liang[1], Yuyao Lu[1], Zikang Deng[3], Honghe Li[3], Mengke Wang[1], M. Jamal Deen[4], Zhiqiu Ye[5], Shuyao Zhou[1], Huayong Yang[1], Honghao Lyu[1,6]*, Jun Chen[7], Kaichen Xu[1], Geng Yang[1,6]*

[1] State Key Laboratory of Fluid Power and Mechatronic Systems, School of Mechanical Engineering, Zhejiang University, Hangzhou 310030, China
[2] Dongfang Electric (Hangzhou) Innovation Institute Co., Ltd., Hangzhou, China
[3] ZJU-UIUC Institute, Zhejiang University, Haining 314400, China
[4] Department of Electrical and Computer Engineering, McMaster University, Hamilton ON L8S 4K1, Canada
[5] Institute of AI for Industries, Chinese Academy of Sciences, Nanjing 211135, China
[6] Zhejiang Key Laboratory of Intelligent Robot for Operation and Maintenance, Hangzhou 310030, China
[7] Department of Bioengineering, University of California, Los Angeles, Los Angeles, CA 90095, USA

Corresponding authors: lvhonghao@zju.edu.cn, yanggeng@zju.edu.cn

This file includes:

Note S1 to 7

Fig. S1 to 24

Table S1 to 3

Supplementary References S1 to 18

Other Supplementary Materials for this manuscript include the following:

Movie S1 to 3

Note S1. Comparison between this work with reported forward design works of TPSs and inverse design work of pressure sensors.

This section provides a comparative analysis of three distinct design methodologies: simulation-driven single-objective inverse design[1], traditional forward design[2-14], and our proposed ML-driven multi-objective inverse design.

Comparison of the initial time cost: Traditional forward design, relying on iterative trial-and-error, typically operates on a weekly timescale (several weeks) to yield a single optimized sensor. Simulation-driven inverse design accelerates data generation to an hourly timescale (several hours) for roughly 100 configurations. Our proposed method incurs an initial time investment intermediate between these two approaches. By leveraging batch processing, we completed the physical fabrication and testing of the 90-sample training set in approximately one week (7 days). This strategy effectively strikes a balance, ensuring high predictive accuracy through physical validation while significantly accelerating the design process.

Comparison of the outcome: The traditional and simulation-driven methods yield one specific sensor with fixed performance profile. Distinct from both, our inverse design approach outputs a comprehensive prediction model and a user interface. This enables the instantaneous recommendation of optimal microstructures for arbitrary, user-defined performance targets (customizable sensitivity, linearity, and detection range), rather than limiting the outcome to a single performance standard.

Comparison of the optimization dimension: Unlike previous works that often focus on a single or two performance metrics, our methodology successfully addresses the multi-objective coupling of three critical performance indicators.

In conclusion, although our initial time investment falls between the simulation-driven inverse-design and traditional approaches, the substantial disparity in outcome justifies our strategy. Unlike both existing paradigms restricted to fixed performance profiles, we effectively transform the experimental effort into a reusable, universal design platform. This drastically high input-output ratio definitively validates the superiority and long-term efficiency of our methodology.

Note S2. Working mechanism of the TPS.

The working mechanism of the TPS is illustrated in **Fig. S1**, which depicts the sensor's response to the application and removal of a dynamic force. PDMS and NBR are pre-charged to a stable charge density and fully separated. PDMS tends to gain electrons while NBR tends to lose electrons. As a result, the surfaces of the two materials acquire equal but opposite charges. The output voltage is the potential difference between the lower (+) and upper (-) electrodes. First, the potential difference is balanced (i). Under a specified force, the compressed distance increases, and the redistribution of surface charges leads to a rise in voltage. When the elastic force, generated by the microstructures, balances the external force, the voltage stabilizes and the corresponding voltage is regarded as the sensor's response to the applied pressure (iii). When the force is removed, the voltage decreases (iv) and returns to the baseline (i). Here, the TPS's response to a specified pressure is clarified based on contact electrification and electrostatic induction.

Note S3. Calculation of D_1^j, D_2^j, and $\bar{\boldsymbol{D}}$.

D_1^j denotes the shortest mathematical distance (also called Euclidian distance) between a targeted microstructure (*j*) and selected microstructures, calculated by **Supplementary Eq. (1)**,

$$D_1^j = \sqrt[2]{\min_{i \in N} \left[\left(\begin{bmatrix} c_i \\ h_i \\ l_i \\ d_i \end{bmatrix} - \begin{bmatrix} c_j \\ h_j \\ l_j \\ d_j \end{bmatrix} \right) \right]^2}, j \in M \qquad (1)$$

where c_i, h_i, l_i, d_i represent crosslinker concentration, height, side length, and density of one selected data point (*i*), and c_j, h_j, l_j, d_j denote crosslinker concentration, height, side length, and density of one targeted data point (*j*), and *N* is the cumulative number of the current selected dataset, and *M* is the cumulative number of the current unselected dataset.

On the other hand, D_2^j denotes the shortest Euclidian distance between the predicted sensing labels of a targeted microstructure (*j*) and those of the selected

microstructures, calculated by **Supplementary Eq. (2)**,

$$D_2^j = \sqrt[2]{\min_{i \in N}\left[\left(\begin{bmatrix} r_i \\ s_i \\ t_i \end{bmatrix} - \begin{bmatrix} r_j \\ s_j \\ t_j \end{bmatrix}\right)^2\right]}, j \in M \tag{2}$$

where r_i, s_i, t_i represent predicted fit labels of a selected data point (i), and r_j, s_j, t_j denote predicted fit labels of a targeted data point (j), and N is the cumulative number of the current selected dataset, and M is the cumulative number of the current unselected dataset.

To represents the degree of design space exploration, $\bar{D}$, meaning the average shortest Euclidian distance of uniformly distributed microstructures in the design space and the selected microstructures, is defined in **Supplementary Eq. (3)**,

$$\bar{D} = \frac{1}{M}\sum_{k=1}^{M} \sqrt[2]{\min_{i \in N}\left[\left(\begin{bmatrix} c_i \\ h_i \\ l_i \\ d_i \end{bmatrix} - \begin{bmatrix} c_k \\ h_k \\ l_k \\ d_k \end{bmatrix}\right)^2\right]} \tag{3}$$

where c_i, h_i, l_i, d_i represent crosslinker concentration, height, side length, and density of a selected data point (i), c_k, h_k, l_k, d_k denote crosslinker concentration, height, side length, and density of a data point (k) in the uniformly distributed design space, and M is the cumulative number of the data points in the design space (M = 247590), and N is the cumulative number of the current selected dataset.

Note S4. Mechanistic insight and surrogate-based performance evaluation of the DP-AL strategy.

While the main text demonstrates the practical efficiency of the DP-AL strategy through physical fabrication, this Note provides a deeper mechanistic analysis of the sampling distribution and performs a comprehensive surrogate-based benchmark to evaluate long-term convergence.

To qualitatively demonstrate the effectiveness of our sampling strategy in balancing exploration and exploitation, we conducted a SHAP analysis on all selected data points (see **Note S6** for details). The data points are visualized in two-dimensional subspaces (e.g., height vs. side length, crosslinker concentration vs. density) with their

SHAP values color-coded (**Fig. S13A**). Notably, the data points are evenly distributed across the ranges of each microstructural parameter, demonstrating the success of diversity-driven exploration. Additionally, regions with larger SHAP values—indicating higher influence on ANN predictions—exhibit locally increased data density. It can be inferred that the uncertainty criterion prioritizes sampling in regions critical for model optimization, even within the globally uniform distribution. Collectively, these findings demonstrate that our sampling strategy achieves an optimal balance between diversity preservation and uncertainty-driven refinement during design space exploration.

To further quantify the convergence speed of the active learning model, ideally, one would perform iterative fabrication for every strategy over multiple loops. However, given the prohibitive time cost of physical experiments, we adopted a surrogate-based benchmarking approach. We utilized our experimentally validated predictor as a ground truth oracle to simulate the iterative learning process (selecting 10 samples per cycle for 9 cycles):

Comparison baselines: We compared our DP-AL strategy (combing input diversity with output gradients) against three established sampling strategies: random sampling, uncertainty sampling, density-weighted uncertainty sampling. Among them, uncertainty Sampling is a standard baseline that prioritizes high-variance regions, a principle central to Bayesian optimization methods[15]. Density-weighted uncertainty is a state-of-the-art strategy balancing uncertainty with input diversity[16-17].

Results and analysis: It should be noted that DP-AL exhibits a slightly higher error in the initial cycles compared to other strategies (**Fig. S13D**). This is because DP-AL prioritizes aggressive exploration of geometrically diverse boundary conditions at the start, sampling the hardest regions first. However, this upfront investment in diversity pays off rapidly. From the 4th cycle onwards, the error of DP-AL drops significantly below all other strategies, achieving the lowest mean relative error (MRE) after 9 cycles.

Why it works (physical explanation): This superiority stems from the high non-linearity of the pressure sensor design space. Standard uncertainty sampling often gets trapped in noisy regions. In contrast, our strategy incorporates performance gradients

that measures distances in the output space. This allows the algorithm to specifically target regions where physical properties change drastically, thus ensuring the rapid convergence of the prediction model.

Note S5. Calculation of MRE for predicted voltages, linearity, and sensitivity.

To evaluate the prediction accuracy of the prediction model, the mean relative error (MRE) between predicted and actual sensing labels is calculated by **Supplementary Eq. (4)**,

$$\mathrm{MRE} = \frac{1}{N}\sum_{i=1}^{N}\left|\frac{predicted^{i} - actual^{i}}{actual^{i}}\right| \tag{4}$$

where N is the number of the evaluation data, $predicted^{i}$ and $actual^{i}$, denote the predicted and actual sensing labels of one data point (i). N is 30 (the number of data points in the test set) when calculating the MRE of predicted fit parameters, voltage, linearity, and sensitivity based on the test set. N is 3 (the number of inverse-designed sensors) when calculating the MRE of predicted sensitivity and linearity of inverse-designed sensors.

Note S6. Working mechanism of SHAP analysis.

Shapley additive explanations (SHAP) is a model explanation algorithm rooted in game theory, designed to assess the contribution of individual features to outputs of any machine learning model. The core concept is derived from Shapley values in game theory, which ensures fair contribution allocation by calculating the marginal effect of each feature on the model output. In SHAP, features are treated as "players," and the model prediction is considered the "total reward." The contribution of each feature is determined by evaluating its impact when incorporated into various feature subsets.

Through the SHAP analysis of the optimal artificial neural network (ANN) model in this work, the SHAP value of each microstructural parameter on a specific sensing label can be calculated. The sensing labels for training the ANN model are the fitting parameters of pressure-voltage curves. Hence the calculated SHAP value means the impact of each microstructural parameter on a fitting parameter. Moreover, the optimal

ANN model can be modified to output voltage values of specified pressure or sensitivity and linearity of specified pressure ranges. Through interpreting the modified optimal ANN model by the SHAP analysis, we can obtain the impact of each microstructural parameter on sensitivity and linearity. The SHAP summary plot indicates the global interpretation of the prediction model over all data points. Furthermore, the SHAP value of every data point can be calculated through averaging the SHAP values of four microstructural parameters, defined in **Supplementary Eq. (5),**

$$\text{SHAP value of points} = \frac{\sum_{i=1}^{N}\sum_{j=1}^{M}\left|\text{SHAP value}^{ij}\right|}{N \times M} \tag{5}$$

where N denotes the number of microstructural parameters of one data point ($N = 4$), M denotes the number of sensing labels of one data point. Through the application of the SHAP analysis, we can systematically quantify the influence of each microstructural parameter, as well as individual data points, on the model outputs.

Note S7 Generality to different elastic materials based on modulus mapping strategy.

The underlying reason to this generality is that our prediction model has thoroughly learned the intrinsic relationship between material modulus and sensing characteristics from the PDMS dataset (which covers a wide range of modulus via varying crosslinker concentrations). Since the mechanical deformation behavior of different elastomers (e.g., Dragonskin vs. PDMS) is fundamentally similar when their modulus is equivalent, the model can apply the modulus-performance rules learned from PDMS to other materials.

To extend the inverse design methodology to arbitrary elastic materials without retraining, we employ a modulus mapping strategy. The mapping is based on a reported formula that fits the relationship between crosslinker concentration and the elastic modulus of PDMS[18], as illustrated in **Supplementary Eq. (6)**,

$$\mathrm{y} = \frac{x - 0.2669}{x + 17.6701} \tag{6}$$

where x and y represent the elastic modulus and crosslinker concentration of PDMS, respectively. To customize sensors using a new elastic material, such as TPU with

modulus of ~3 MPa, the equivalent crosslinker concentration is calculated as 0.132 based on **Supplementary Eq. (6)**, which is then applied as a fixed constraint in **Eq.5**. This allows the NSGA-II algorithm to search for optimal geometries (height, density, side length) specific to that modulus.

Experimental verification: To validate this strategy, we input the performance targets of TPS-3 along with the respective modulus constraints of new materials (TPU, ~3 MPa; Dragonskin, ~1 MPa) into the software. Based on these inputs, the software recommended the same optimized parameter set (0.91/0.91/0.1) (**Fig. S23 A-B**). The modulus of TPU (~3 MPa) falls within the range of our PDMS database (1.4-3.7 MPa, calculated based on **Supplementary Eq. (6)**). Consequently, the predicted pressure-voltage curve matches the experimental results well, validating the accuracy of the modulus mapping (**Fig. S23 C**). Additionally, the modulus of Dragon Skin (~1 MPa) is lower than the database range. This led to a noticeable degradation in prediction fidelity. The predicted curve exhibits limited linearity and voltage offsets (**Fig. S23 D**). However, crucially, the experimental Dragon Skin sensor still exhibits the desired high linearity.

In summary, while prediction accuracy is highest for materials within the database range, our inverse design strategy remains robust enough to guide the fabrication of high-performance sensors even for materials outside the range.

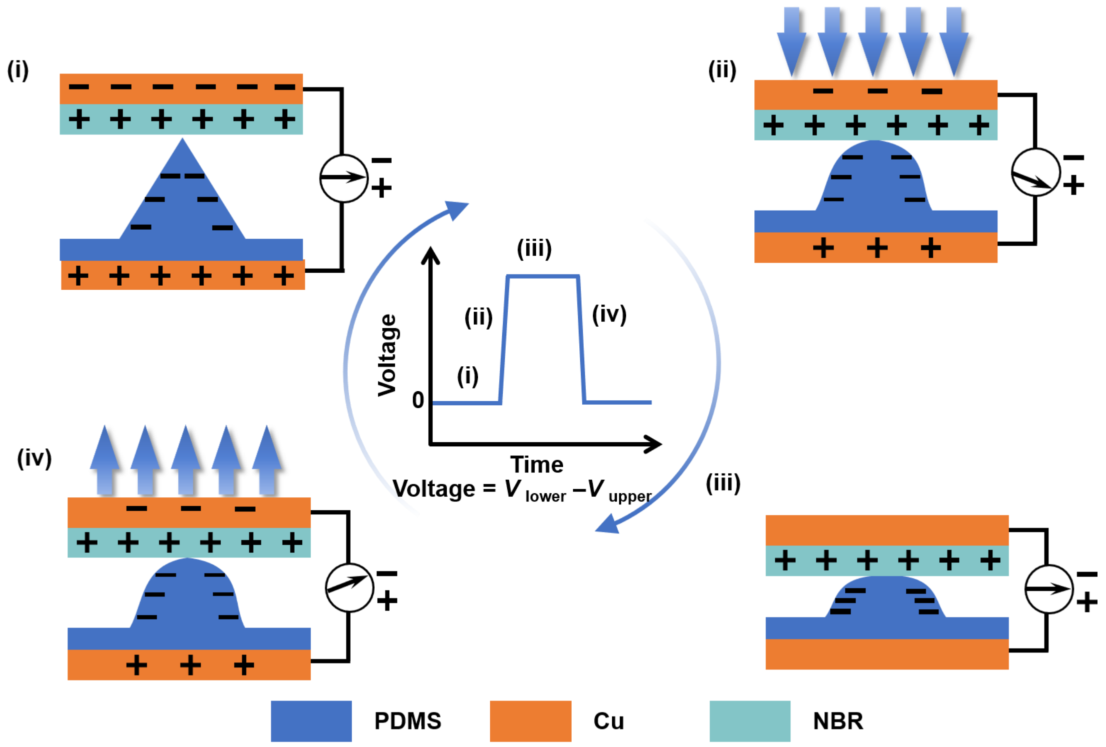

Fig. S1. Working mechanism of the TPS. The schematic illustration depicts the sensor's response to the application and removal of a dynamic force. PDMS and NBR are pre-charged to a stable charge density and fully separated. PDMS tends to gain electrons while NBR tends to lose electrons. As a result, the surfaces of the two materials acquire equal but opposite charges. The output voltage is the potential difference between the lower (+) and upper (-) electrodes. First, the potential difference is balanced (i). Under a specified force, the compressed distance increases, and the redistribution of surface charges leads to a rise in voltage. When the elastic force, generated by the microstructures, balances the external force, the voltage stabilizes and the corresponding voltage is regarded as the sensor's response to the applied pressure (iii). When the force is removed, the voltage decreases (iv) and returns to the baseline (i). Here, the TPS's response to a specified pressure is clarified based on contact electrification and electrostatic induction.

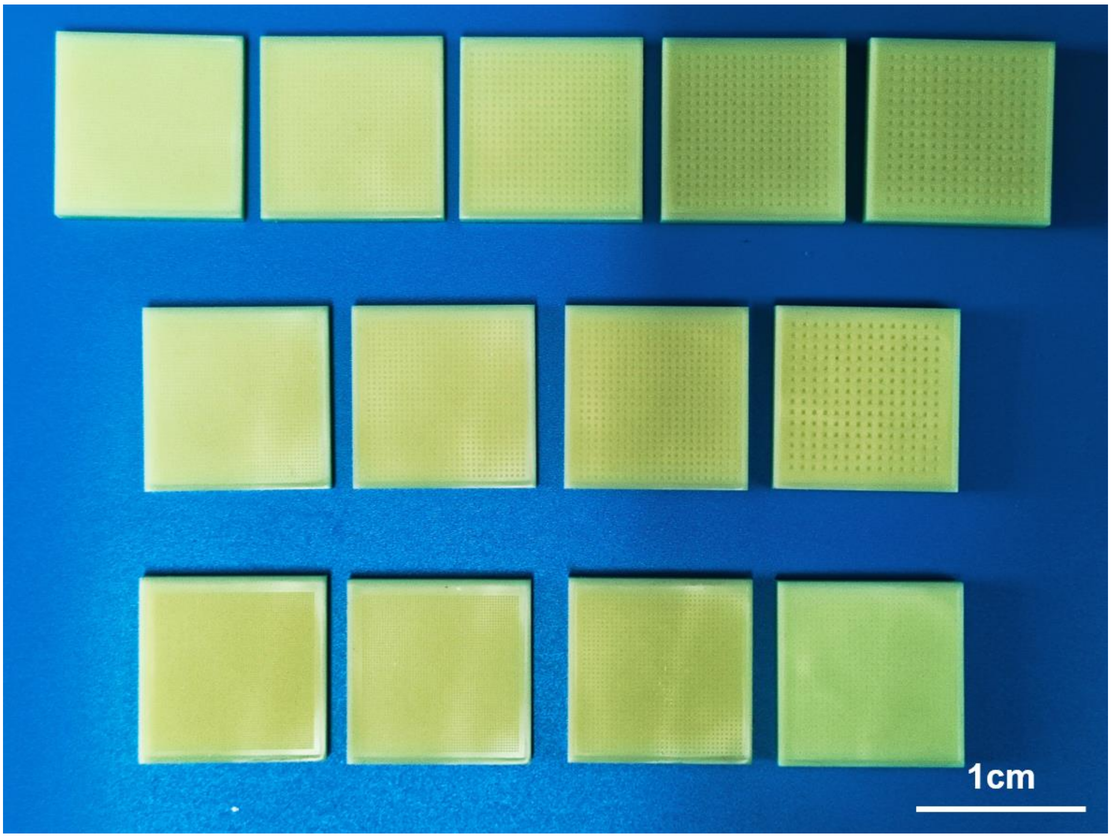

Fig. S2. Photographs of 3D-printed microstructure molds with different parameters. (scale bar, 1 cm). The templates were fabricated by a high-precision photocurable 3D printing machine with a resolution: 30 μm in XY plane, 5 μm in Z axis. PDMS films with different microstructures can be fabricated based on the templates.

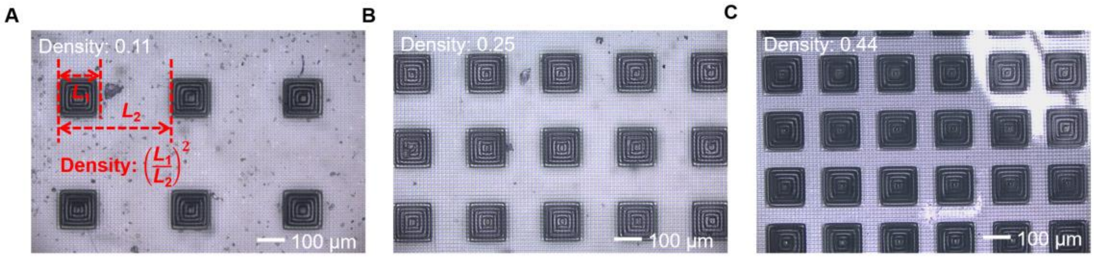

Fig. S3. Top-view optical microscope images of microstructures with varying density. A-C. Density, 0.11 (A), density, 0.25 (B), density, 0.44 (C), scale bar, 100 μm. Density is defined as the square of the ratio of side length to the sum of side length and spacing, as illustrated in (A).

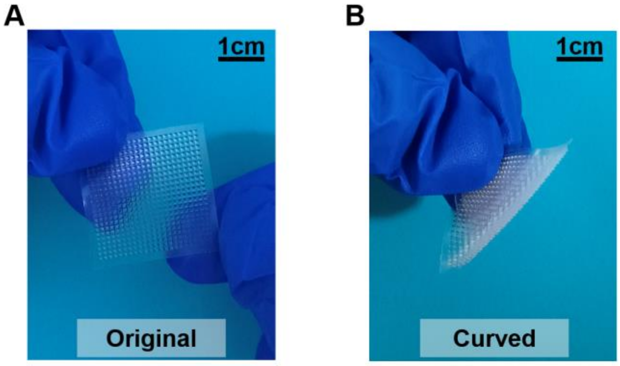

Fig. S4. Photographs of fabricated PDMS films with microstructures in original and curved states, showing great flexibility (scale bar, 1 cm). A. A PDMS film at the original state. B. A PDMS film at the curved state.

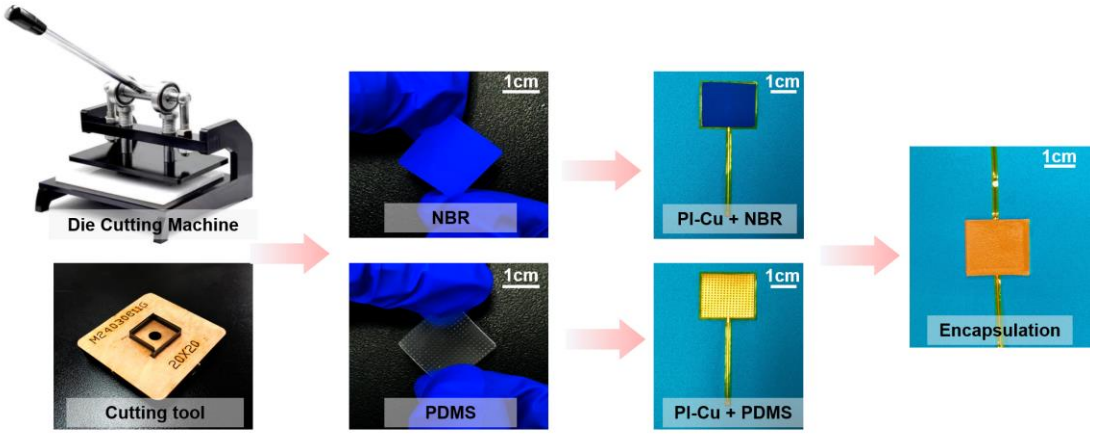

Fig. S5. Integration process of the TPS. The microstructures with varying crosslinker concentration, height, side length, and density can be obtained by the 3D printing-enabled technique. NBR films and patterned PDMS films were cut by a die cutting machine, while PI-Cu films were customized flexible printed circuit boards. Next, a TPS is fabricated by sequentially integrating two PI-Cu films, NBR films, and patterned PDMS films in a layered structure. The size of Cu, NBR films, and patterned PDMS films is 2 cm×2 cm.

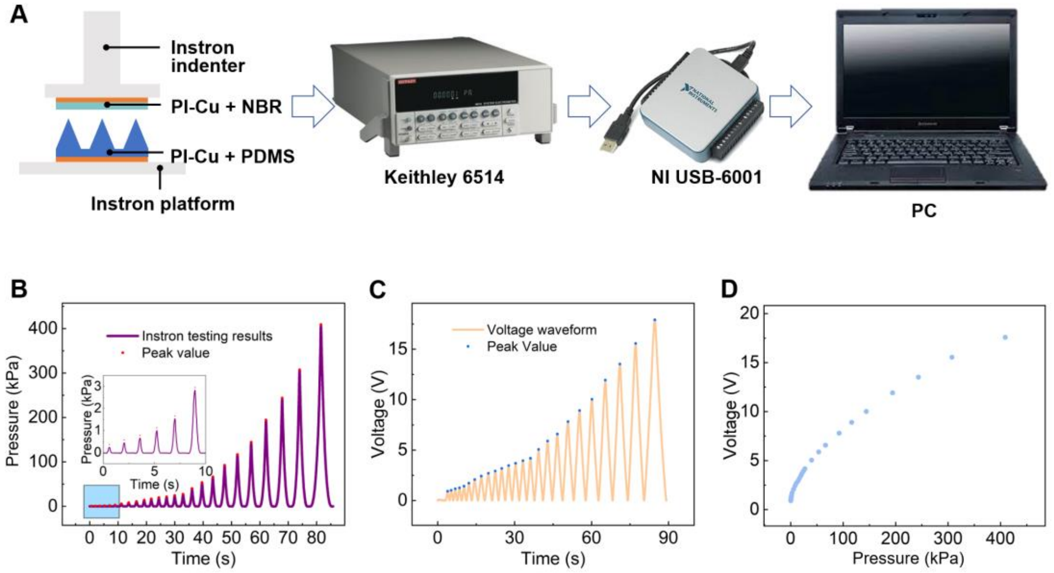

Fig. S6. Calibration and signal processing of the TPS. A. Force testing platform and signal acquisition process. B. Dynamic force measurement: raw force curves (solid lines) and extracted peak values (circles). Inset shows magnified view of low-force region with corresponding peak detection. C. Sensor output characterization: voltage response curves (solid lines) and extracted peak values (circles). D. Sensor calibration: Pressure-voltage relationship derived from synchronized force and voltage measurements.

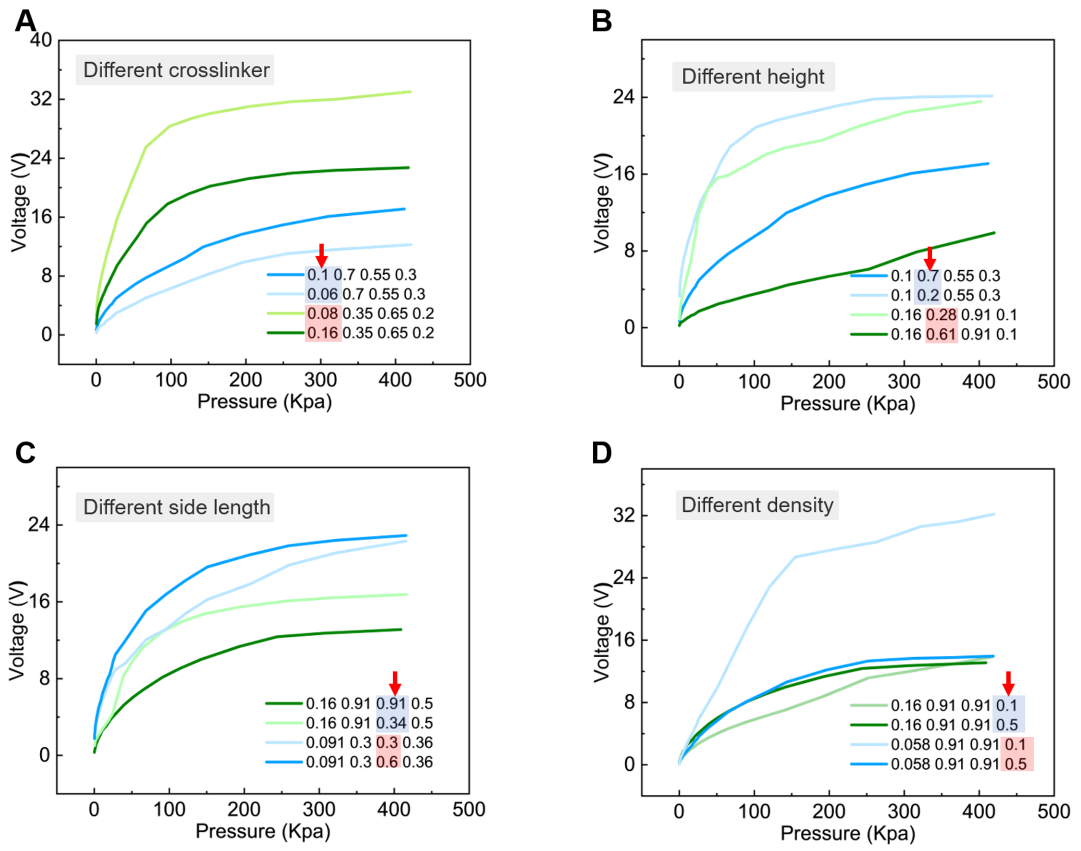

Fig. S7. Influence of microstructures on pressure sensing characteristics. A-D. Pressure-voltage curves of sensors with different parameters, indicating that crosslinker concentration (A), height (B), side length (C), and density (D) are influential parameters on sensing performance. The microstructure labels in each figure are ordered as following, crosslinker concentration, height (mm), side length (mm), and density. There are two comparison groups highlighted in blue and red in each figure.

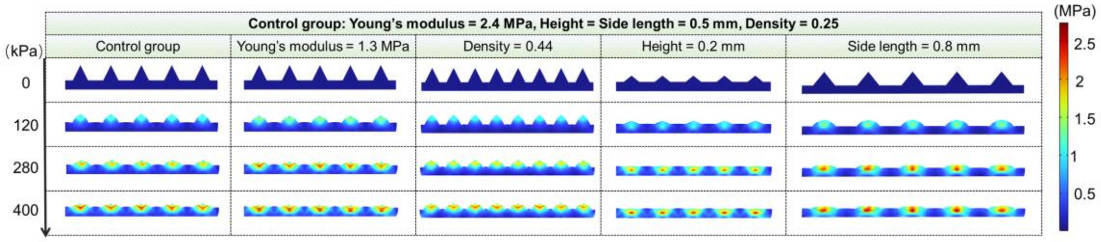

Fig. S8. Simulated deformation of microstructures under pressures increasing from 0 to 400 kPa for different Yong's modulus, density, height, and side length. This indicates that the design variables significantly influence the deformation process of microstructures, confirming the rationale of selecting them as the design variables.

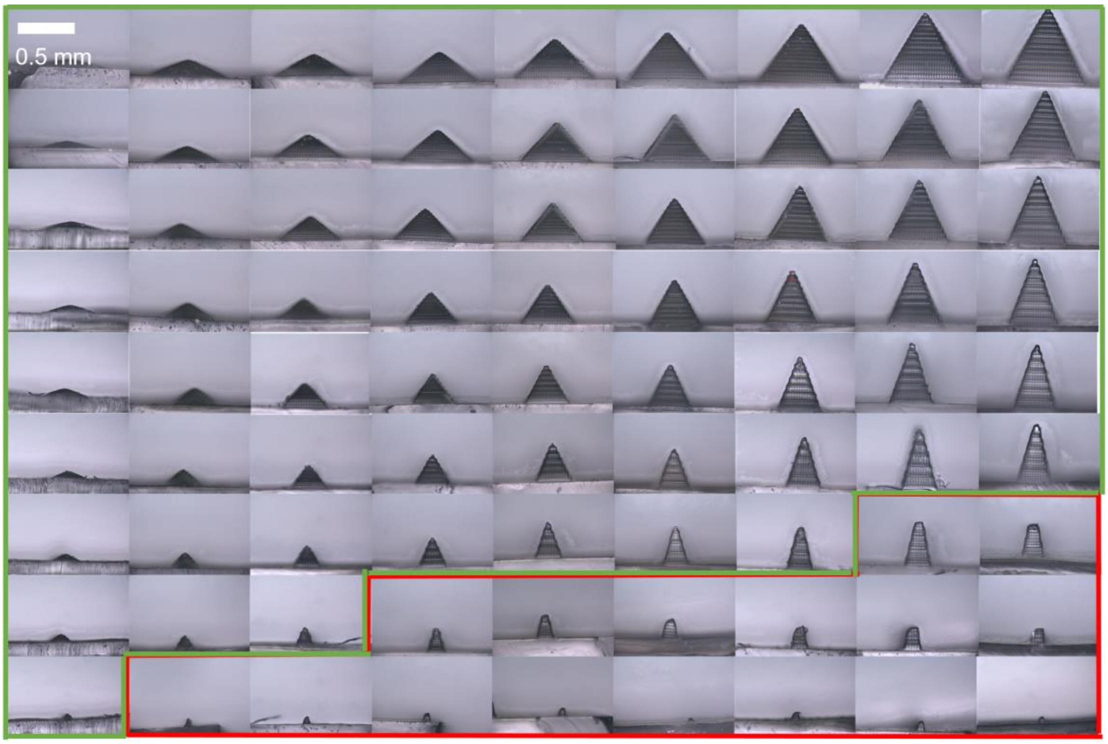

Fig. S9. Side-view optical microscope images of 3D-printed microstructures used for training the support vector machine (scale bar, 0.5 mm). The microstructures in the red frame are failed cases (defective trapezoids), while those in the green frame are successful cases (intact triangles).

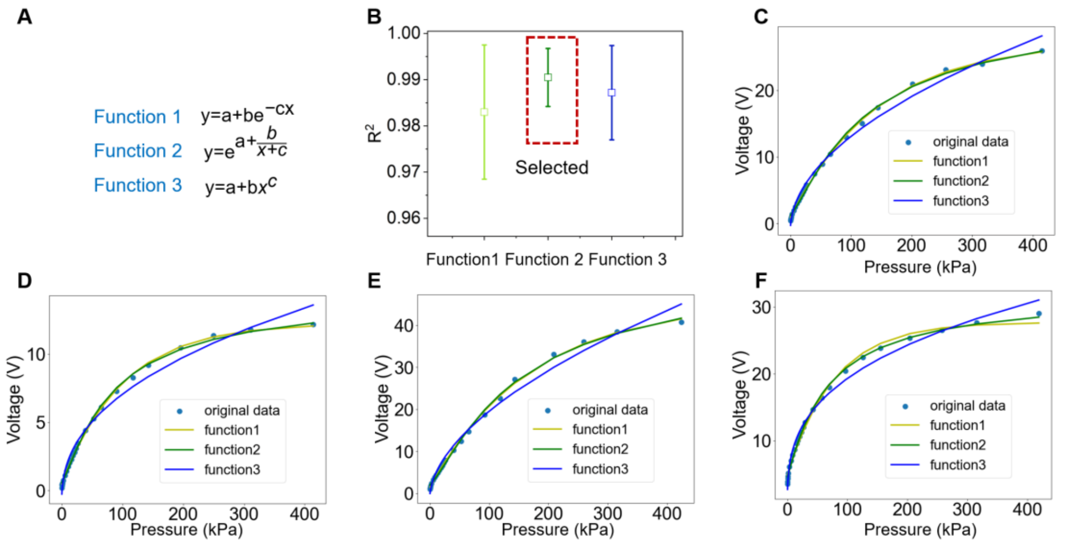

Fig. S10. Using various mathematical equations to fit pressure–voltage curves. A. Different mathematical equations to fit pressure–voltage curves. B. Coefficients of determination (R^2) of three fitting equations. The box plot shows the 25th to 75th percentiles with the median at the center line, and whiskers extending to the 5th and 95th percentiles (n = 11). The function-2 exhibits the superb fitting effect, thus selected to model the pressure-voltage curves. C-F. Original and fitting curves of four different sensors.

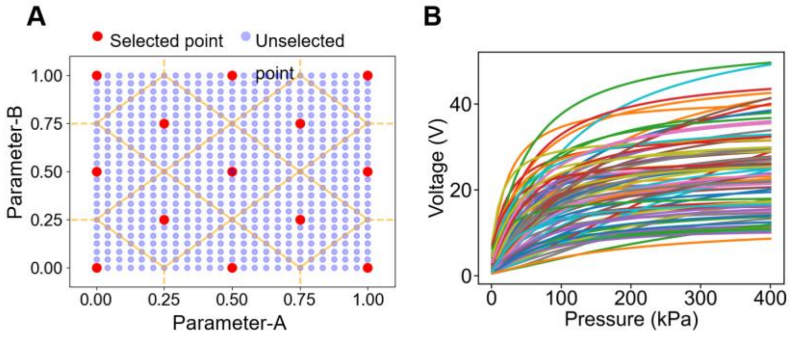

Fig. S11. Active learning-guided fabrication of TPSs. A. Schematic diagram of the acquisition function in the initial active learning phase, ensuring the diversity of selected data points. B. Pressure-voltage sensing curves of selected microstructures after 9 active learning loops, exhibiting diverse sensing performance.

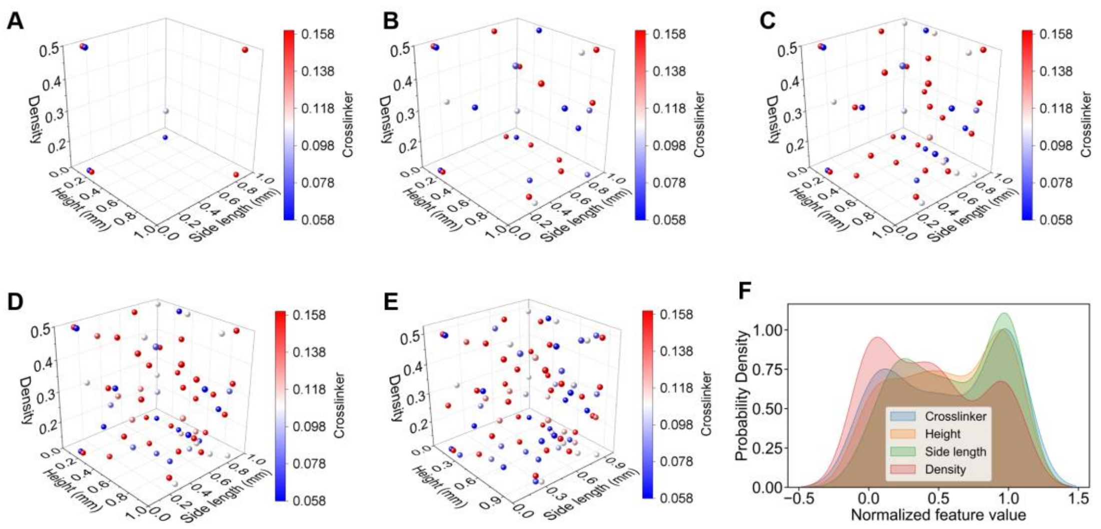

Fig. S12. Distribution of selected microstructures in the design space at various active learning stages. A. After 1st rounds. B. After 3rd rounds. C. After 5th rounds. D. After 7th rounds. E. After 9th rounds. The active learning algorithm progressively explores the design space. F. Probability density distribution of selected microstructures after 9 active learning loops, indicating relatively uniform distribution in the design space. The uneven region is explained in **Note S4**.

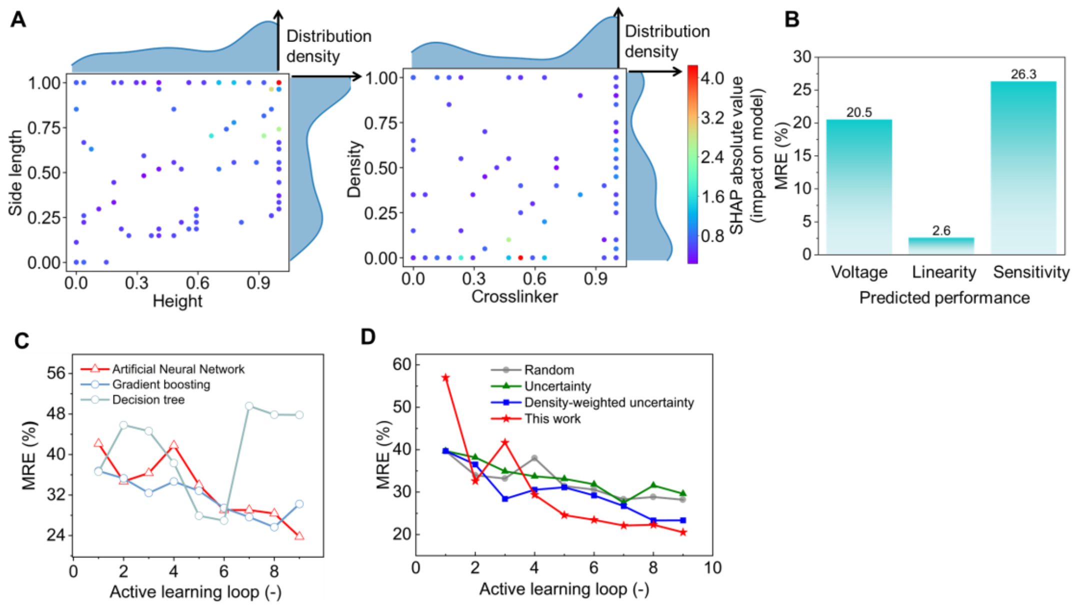

Fig. S13. Evaluation of active learning algorithm from mechanistic insight, the selection of prediction models, and surrogate-based convergence benchmarking. A. Selected microstructures visualized in the subspaces, with marginal density distributions and color-coded SHAP values of points (see **Note S6** for details). B. Evaluation of the prediction model's accuracy on sensing performance with the test set (n = 30). MRE (see **Note S5** for details) of predicted voltage at 10, 50, 100, 200, 400 kPa and MRE of predicted sensitivity and linearity at 0-10, 0-50, 0-100, 0-200, 0-400 kPa. The MRE for predicted voltage, linearity, and sensitivity across the whole pressure range achieves 21%, 2.6%, and 26%, indicating superb accuracy. C. MRE of predicted fit labels for different prediction models during the active learning loops. The ANN indicates the superb accuracy compared with other ML models. D. Quantitative benchmarking of data-efficiency and convergence speed across different active learning strategies.

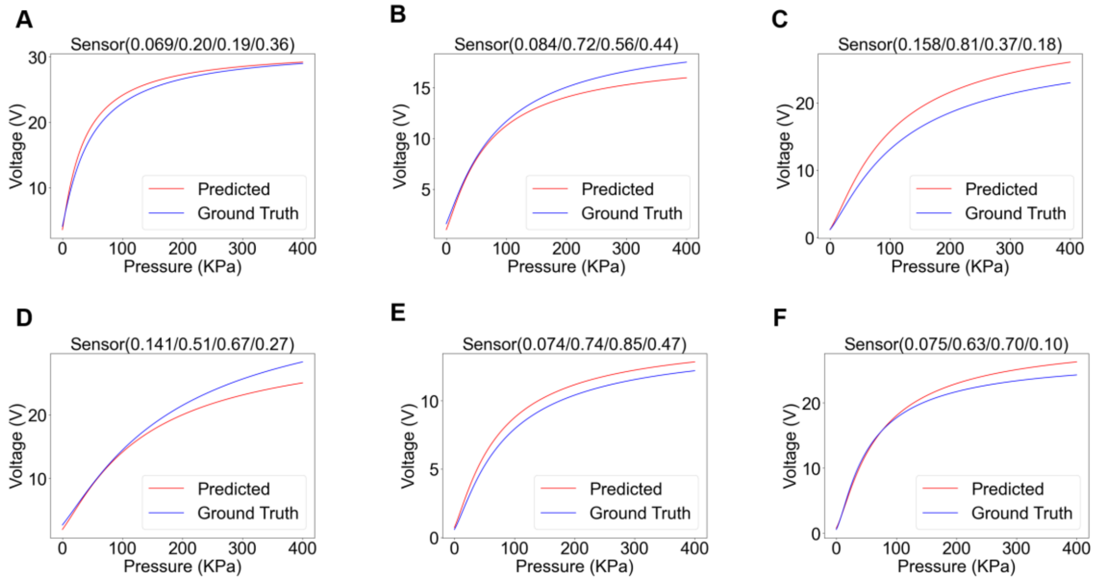

Fig. S14. Measured and predicted sensing curves of TPSs in the test set, indicating high accuracy of the optimal ANN model. A-F. Predicted and measured (ground truth) pressure-voltage curves of pressure sensors in the test set. The microstructure labels in each figure are ordered as following, crosslinker concentration, height (mm), side length (mm), and density.

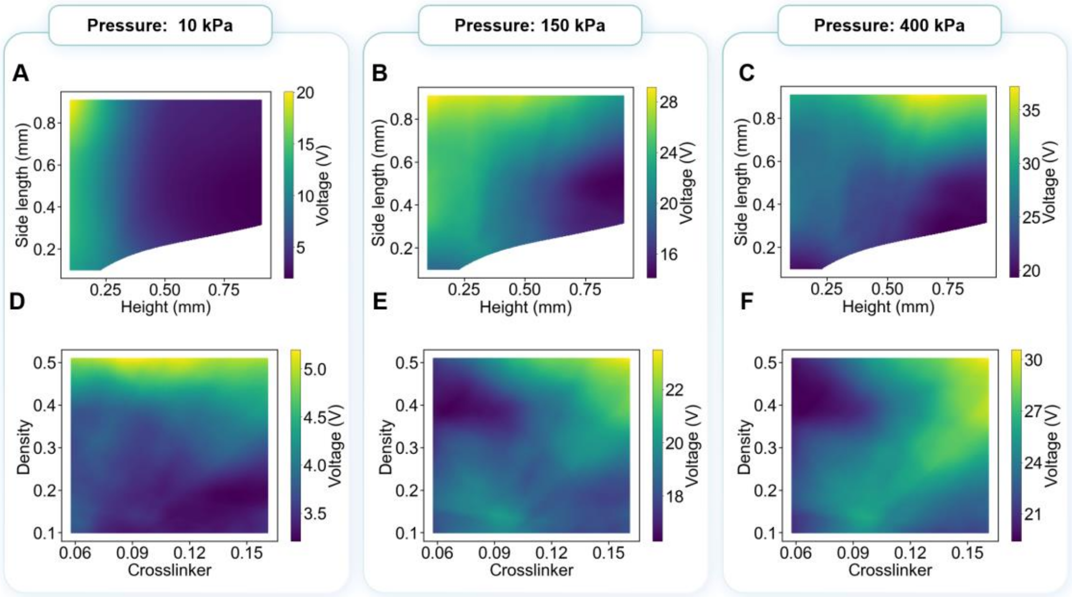

Fig. S15. Predictive mapping between microstructures and output voltages of the TPS at different pressures. A-C Predicted voltages (color-coded) of the height-side length subspace at pressures of 10 kPa, 150 kPa, and 400 kPa. D-F. Predicted voltages (color-coded) of the crosslinker-density subspace at pressures of 10 kPa, 150 kPa, and 400 kPa. The default crosslinker concentration, height, side length, and density of the above figures are 0.11, 0.5 mm, 0.5 mm, and 0.1.

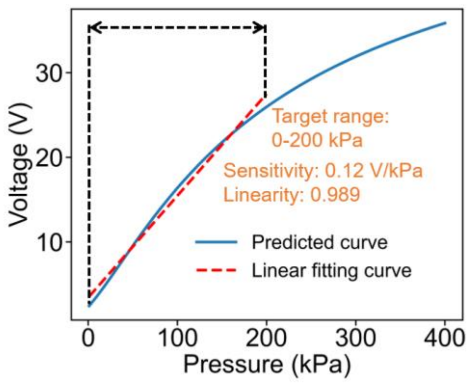

Fig. S16. Calculation method of sensitivity and linearity within the specified detection range. After inputting microstructure parameters to the prediction model, the model outputs predicted pressure-voltage curves. Then the sensitivity and linearity within targeted detection ranges can be calculated based on the predicted curves, thus enabling the prediction of linearity and sensitivity of any detection range.

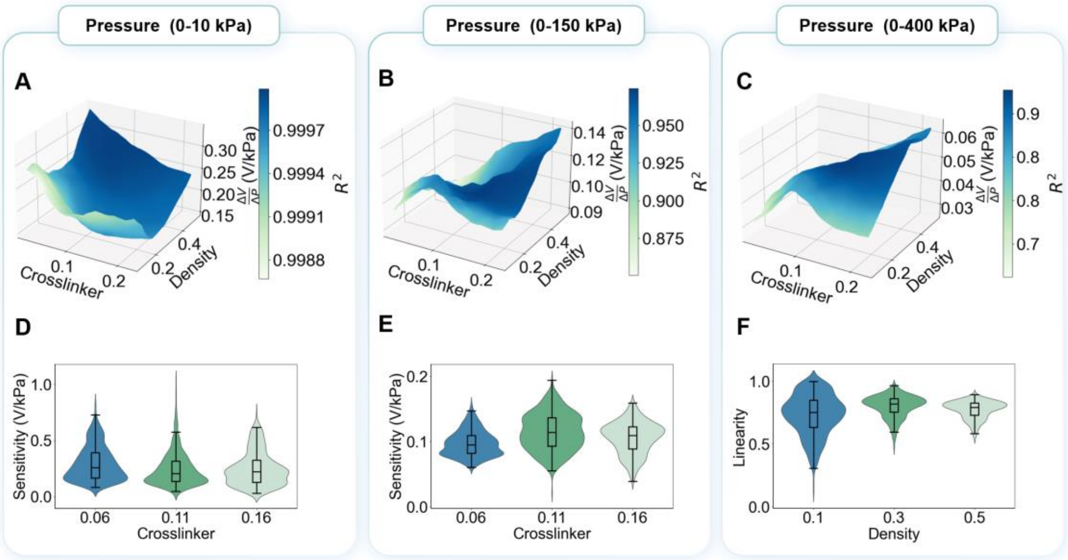

Fig. S17. Predictive mapping between microstructures and sensitivity, linearity across different detection ranges. A-C. Predictive sensitivity and linearity of the crosslinker-density subspace across detection ranges: 0-10 kPa (A), 0-150 kPa (B), and 0-400 kPa (C). D-E. Violin plots illustrating predictive sensitivity for pressure ranges of 0-10 kPa (D) and 0-150 kPa (E), with crosslinker concentrations of 0.06, 0.11, and 0.16. F. Violin plot illustrating the linearity predictions for pressure ranges of 0–400 kPa, with density of 0.1, 0.3, and 0.5. The embedded box plots within each violin plot indicates the 25th and 75th percentiles with the median represented by the center line. Whiskers extend to 1.5× IQR from the box (n = 2751 (D-E), n = 2358 (F)).

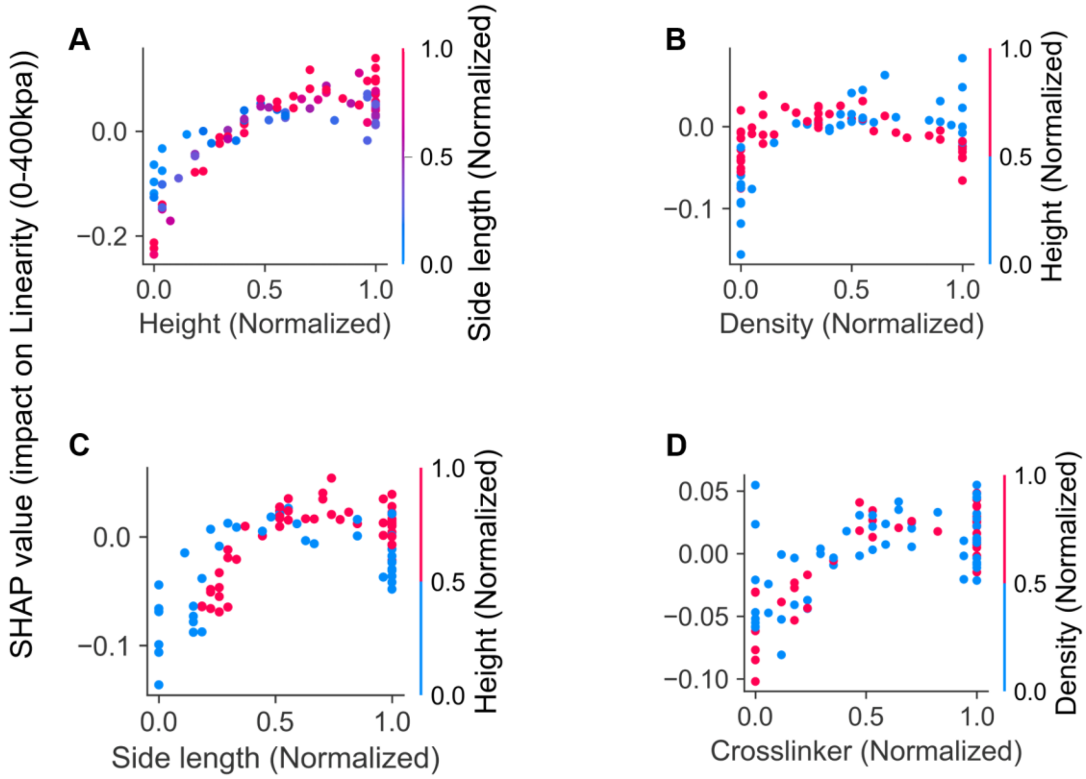

Fig. S18. SHAP independence plots for linearity (0-400 kPa). A-D. Relationships between SHAP values and height (A), density (B), side length (C), and crosslinker (D), with colors indicating the value of most strongly correlated factor. For example, the height has a positive effect on linearity (0-400 kPa). When the normalized height is below 0.5, a smaller side length is beneficial for improving linearity (0-400 kPa). However, when the normalized height exceeds 0.5, a larger side length is more favorable. These observations suggest that microstructures that are both low and thin, or tall and thick, are advantageous for enhancing linearity (0-400 kPa).

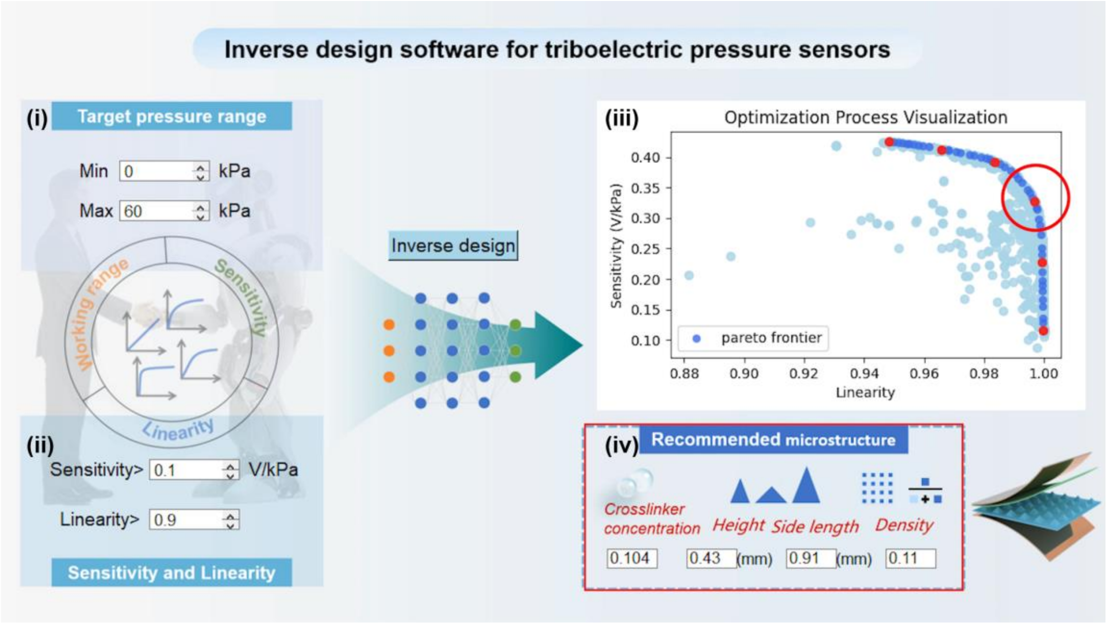

Fig. S19. NSGA-II-based inverse design software for customizable TPSs. (i) User-defined target pressure range. (ii) Performance targets for sensitivity and linearity. (iii) Optimization process visualization showing the Pareto frontier, with six uniformly distributed representative points (red circle indicates the predicted performance of the final recommended microstructure). (iv) The optimal microstructure output by the software. The software automatically generates the Pareto-optimal solutions that maximizes both sensitivity and linearity, enabling selection of microstructures based on application-specific priorities (i.e., users select the most suitable microstructure from the representative points).

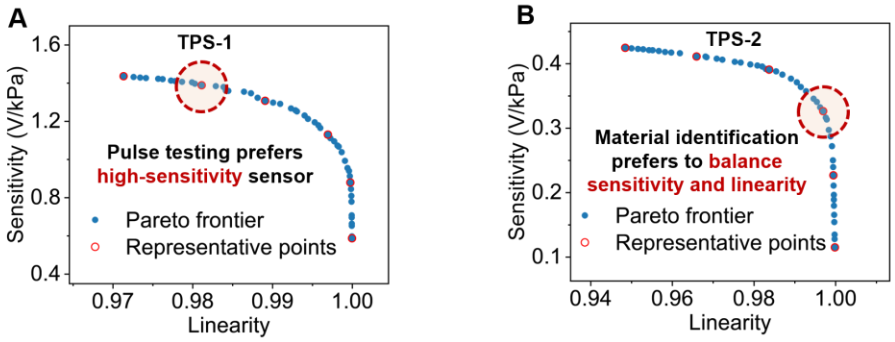

Fig. S20. Pareto frontier obtained from the inverse design UI. A-B. Predicted pareto frontier for pulse testing (A), material identification (B).

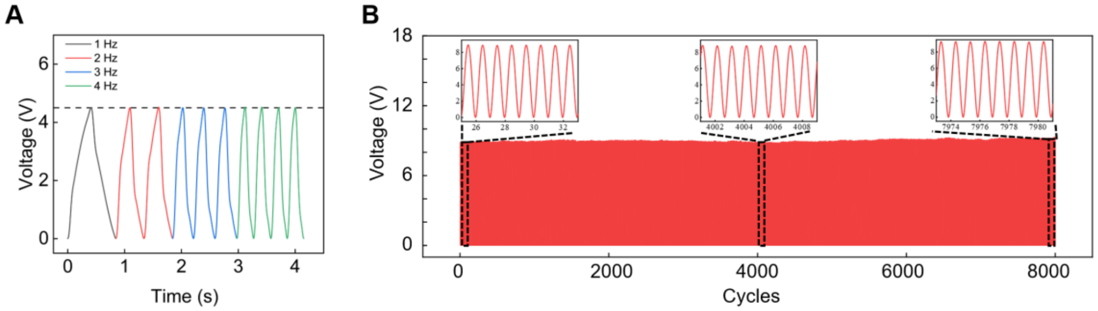

Fig. S21. Frequency response (1-4 Hz) and durability tests (8000 cycles) of the TPS. A. Frequency response test at pressure of 50 kPa. B. Durability test lasting for 8000 cycles at pressure of 100 kPa.

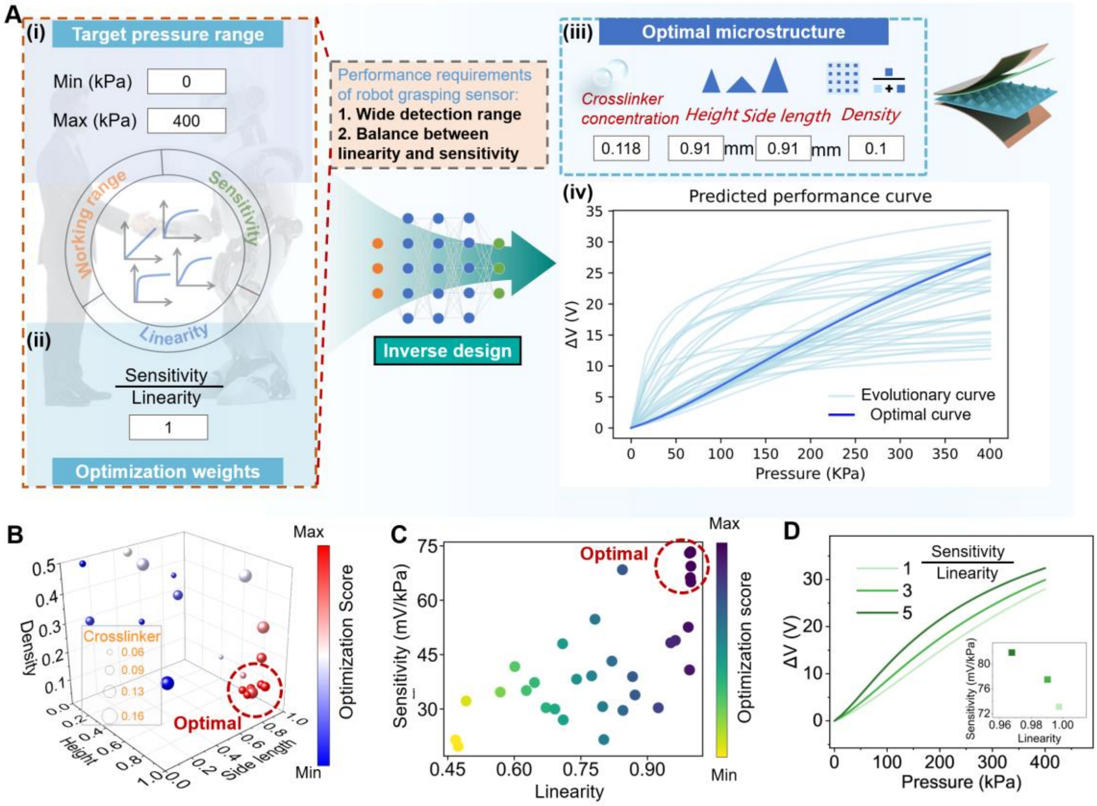

Fig. S22. Inverse design process based on Bayesian Optimization (BO) algorithm. A. Inverse design process for designing a robot grasping sensor, consisting of (i) targeted pressure ranges, (ii) optimization weights, (iii) optimal structures, and (iv) predicted performance curves. B-C. Microstructural parameters (B) and predicted sensitivity and linearity during the optimization process (C). D. Optimal sensing curves corresponding to optimization weight of 1, 3, and 5. The optimal solution is a subset of the Pareto front generated by NSGA-II, demonstrating that NSGA-II performs better than BO in the optimization problem.

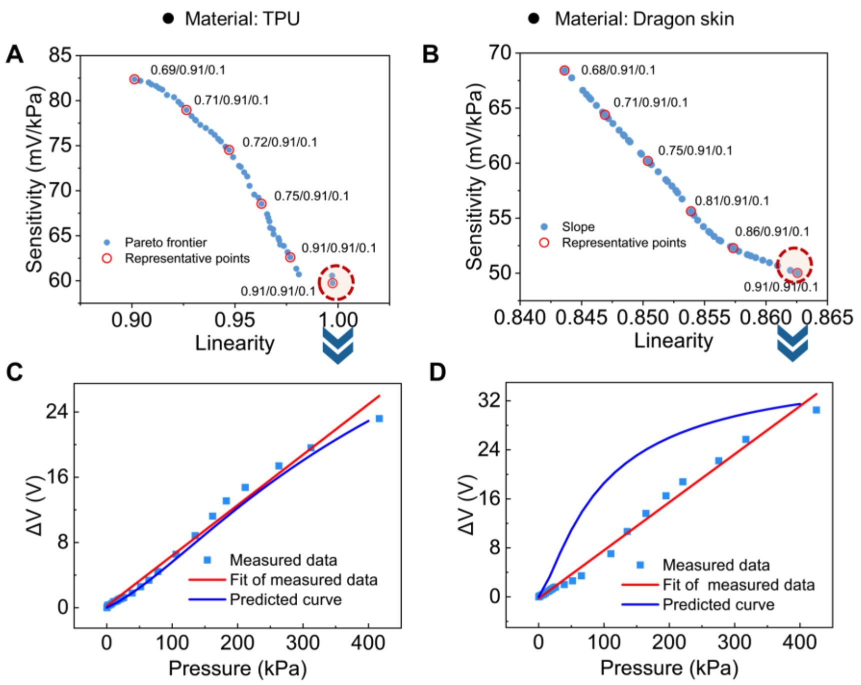

Fig. S23. Generalizability demonstration of inverse design to different materials. A-B. Pareto frontier obtained from the inverse design UI for TPU-TPS (A), Dragonskin-TPS (B). C-D. Predicted, measured, and fit sensing curves for TPU-TPS (C), Dragonskin-TPS (D).

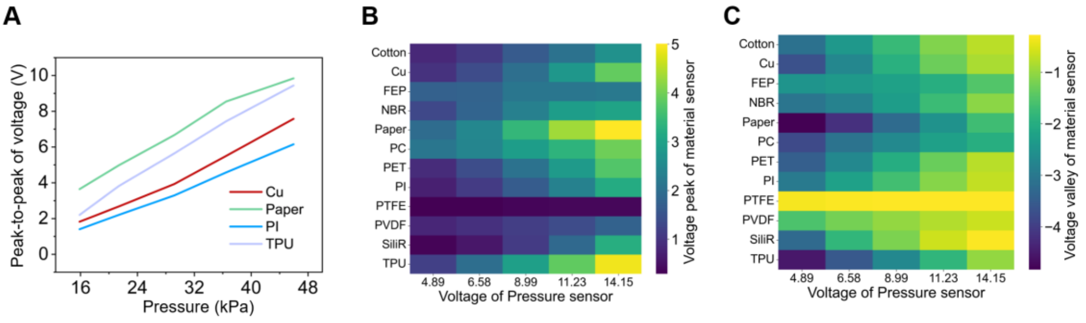

Fig. S24. Material identification based on the inverse-designed material sensor (single-electrode TPS) and the TPS (double-electrode). A. Relationships between peak-to-peak voltages and pressures for Cu, Paper, PI, and TPU, exhibiting great linearity. This demonstrates the inverse design platform is adapt to designing the single-electrode TPS. B. Peak voltage values along with the pressure sensor's output for 12 materials. C. Valley voltage values along with the pressure sensor's output for 12 materials.

Table S1. Comparison between inverse-designed TPSs and recently reported TPSs. The three TPSs in this work are not all pressure sensors that the inverse design platform can develop. TPSs with higher performance can be developed through inputting more superb performance requirements to the inverse design platform.

Design approach	Detection range (kPa)	Sensitivity (V/kPa)	Thickness	Reference
Empirical forward design	5-50	0.127	<2 mm	Ref[2]
	0-20<br>20-100	0.55<br>0.2		Ref[3]
	0-1<br>1-20	1.04<br>0.19		Ref[4]
	0-26<br>26-120	0.75<br>0.19		Ref[5]
	0-0.25<br>0.25-1.5	0.433<br>0.34		Ref[6]
	0-200	0.177		Ref[7]
	0-10<br>10-30<br>30-100	0.75<br>0.4<br>0.05		Ref[8]
	0-75<br>75-250	0.071<br>0.011		Ref[9]
	0-10<br>10-200	0.076<br>0.028		Ref[10]
	0.1-2<br>2-60	7.99<br>0.46		Ref[11]
	0-10<br>10-35	5.84<br>1		Ref[12]
	0-7.13<br>7.13-453.83	8.5<br>0.11	> 2 mm	Ref[13]
	5-1230	0.023	> 1 cm	Ref[14]
Data-driven inverse Design (This work)	0-10	1.229	< 2 mm	TPS-1
	0-60	0.308		TPS-2
	0-400	0.073		TPS-3

Table S2. 90 data points collected from 9 loops of active learning. A total of 9 loops of active learning were executed, and 90 pressure sensors were fabricated cumulatively.

Index	Loop	Microstructure parameters				Sensing labels		
		Crosslinker	Height (mm)	Side length (mm)	Density	a	b	c
1	1	0.1	0.49	0.55	0.3	3.409	-19.03	6.62
2	1	0.058	0.1	0.1	0.1	3.731	-2.043	1.025
3	1	0.16	0.1	0.1	0.5	2.562	-5.272	3.286
4	1	0.058	0.13	0.1	0.5	3.501	-2.053	1.184
5	1	0.16	0.13	0.1	0.1	3.092	-1.032	0.856
6	1	0.058	0.91	0.91	0.1	3.69	-8.881	2.016
7	1	0.058	0.91	0.91	0.5	2.833	-7.765	2.233
8	1	0.16	0.91	0.91	0.1	3.019	-20.07	6.482
9	1	0.16	0.91	0.91	0.5	2.81	-8.023	2.864
10	1	0.058	0.1	0.91	0.1	3.272	-2.069	1.124
11	2	0.16	0.88	0.31	0.12	3.257	-6.616	2.327
12	2	0.16	0.28	0.91	0.1	3.151	-2.714	0.905
13	2	0.16	0.91	0.37	0.46	2.553	-5.933	1.775
14	2	0.07	0.67	0.52	0.1	2.946	-4.058	1.07
15	2	0.16	0.61	0.91	0.1	3.438	-5.573	1.519
16	2	0.16	0.13	0.91	0.36	3.341	-4.396	2.243
17	2	0.106	0.91	0.34	0.1	3.125	-4.015	1.218
18	2	0.082	0.91	0.91	0.1	3.724	-8.487	2.241
19	2	0.16	0.1	0.79	0.12	3.306	-1.774	1.092
20	2	0.058	0.34	0.91	0.5	2.88	-3.778	1.237
21	3	0.16	0.13	0.64	0.5	3.304	-2.913	1.613
22	3	0.058	0.4	0.22	0.34	3.2	-2.4	1.12
23	3	0.082	0.91	0.88	0.32	2.816	-8.284	2.846

24	3	0.076	0.76	0.28	0.5	2.847	-2.863	1.25
25	3	0.112	0.88	0.79	0.5	2.612	-6.449	2.315
26	3	0.058	0.79	0.91	0.24	3.231	-6.696	2.039
27	3	0.106	0.1	0.19	0.32	2.393	-3.719	1.431
28	3	0.16	0.85	0.67	0.14	3.573	-33.18	7.802
29	3	0.058	0.91	0.61	0.36	3.076	-9.732	3.65
30	3	0.16	0.91	0.91	0.34	2.504	-6.067	2.617
31	4	0.13	0.91	0.4	0.3	3.553	-10.24	2.673
32	4	0.16	0.73	0.55	0.14	4.008	-12.47	3.371
33	4	0.16	0.88	0.88	0.24	3.212	-36.95	10.418
34	4	0.112	0.91	0.91	0.1	4.306	-49.03	11.955
35	4	0.112	0.91	0.55	0.2	3.478	-13.61	4.563
36	4	0.16	0.28	0.25	0.32	3.52	-8.689	4.31
37	4	0.058	0.91	0.46	0.24	2.843	-5.348	1.735
38	4	0.112	0.43	0.91	0.5	3.42	-7.184	2.976
39	4	0.16	0.34	0.91	0.3	3.466	-9.422	3.569
40	4	0.106	0.91	0.7	0.14	3.98	-38.36	9.617
41	5	0.16	0.58	0.28	0.46	3.421	-7.006	2.247
42	5	0.16	0.55	0.91	0.24	2.608	-9.227	3.464
43	5	0.07	0.61	0.91	0.1	3.757	-5.003	1.43
44	5	0.16	0.49	0.22	0.2	3.442	-6.009	2.207
45	5	0.058	0.37	0.91	0.1	3.528	-5.63	1.832
46	5	0.106	0.1	0.91	0.5	3.146	-5.177	2.669
47	5	0.154	0.31	0.22	0.1	3.282	-2.017	0.981
48	5	0.16	0.37	0.58	0.1	2.77	-3.731	1.283
49	5	0.16	0.64	0.67	0.32	3.405	-27.91	9.265
50	5	0.16	0.49	0.79	0.44	3.435	-15.06	5.327
51	6	0.16	0.43	0.52	0.38	3.88	-9.916	3.262
52	6	0.16	0.25	0.37	0.46	3.869	-4.974	2.255

53	6	0.088	0.19	0.34	0.24	4.012	-4.454	1.628
54	6	0.124	0.73	0.73	0.18	4.061	-16.74	5.147
55	6	0.076	0.52	0.52	0.1	2.966	-7.417	2.04
56	6	0.142	0.13	0.28	0.46	3.479	-4.883	2.489
57	6	0.13	0.37	0.49	0.3	3.182	-5.013	2.117
58	6	0.16	0.91	0.52	0.4	3.502	-9.029	3.038
59	6	0.058	0.13	0.31	0.16	3.415	-2.047	1.088
60	6	0.154	0.67	0.91	0.14	4.12	-9.541	2.595
61	7	0.16	0.58	0.31	0.32	3.104	-5.605	1.734
62	7	0.13	0.55	0.25	0.26	3.297	-7.66	2.634
63	7	0.118	0.43	0.88	0.1	3.699	-3.913	1.2
64	7	0.082	0.52	0.28	0.16	3.88	-4.429	1.491
65	7	0.112	0.58	0.34	0.5	3.651	-6.808	2.619
66	7	0.16	0.73	0.91	0.28	3.567	-18.62	6.182
67	7	0.13	0.43	0.22	0.32	3.718	-5.654	2.095
68	7	0.094	0.7	0.7	0.12	3.499	-7.693	2.437
69	7	0.112	0.1	0.91	0.26	3.484	-1.234	0.788
70	7	0.16	0.91	0.52	0.22	3.448	-10.15	3.175
71	8	0.07	0.88	0.31	0.1	2.801	-6.727	1.858
72	8	0.16	0.16	0.61	0.28	3.543	-2.748	1.396
73	8	0.076	0.85	0.91	0.5	2.554	-7.172	2.273
74	8	0.076	0.22	0.1	0.1	3.308	-1.993	1.391
75	8	0.07	0.85	0.76	0.5	2.477	-6.512	2.06
76	8	0.058	0.82	0.55	0.5	3.4	-5.505	1.65
77	8	0.064	0.91	0.34	0.24	3.338	-7.546	2.002
78	8	0.124	0.88	0.31	0.5	3.199	-4.528	1.478
79	8	0.088	0.43	0.25	0.1	3.334	-4.002	1.27
80	8	0.16	0.25	0.91	0.48	3.579	-3.591	1.762
81	9	0.094	0.91	0.64	0.38	2.698	-7.465	2.436

82	9	0.142	0.91	0.88	0.24	2.517	-16.36	5.312
83	9	0.154	0.1	0.1	0.26	2.935	-2.036	1.805
84	9	0.094	0.43	0.91	0.28	3.603	-5.223	1.938
85	9	0.076	0.91	0.52	0.44	2.91	-7.369	2.918
86	9	0.124	0.67	0.91	0.1	4.148	-19.57	6.048
87	9	0.118	0.34	0.91	0.22	3.698	-5.38	2.026
88	9	0.058	0.55	0.91	0.24	3.546	-6.724	2.102
89	9	0.082	0.25	0.46	0.5	3.486	-3.655	1.776
90	9	0.064	0.58	0.25	0.1	2.891	-3.335	1.027

Table S3. Test set for evaluating the accuracy of the prediction model. The test set consists of 30 randomly collected data points from the design space.

Index	Microstructure parameters				Sensing labels		
	Crosslinker	Height (mm)	Side length (mm)	Density	a	b	c
1	0.063	0.17	0.89	0.17	3.674	-3.168	1.175
2	0.063	0.48	0.28	0.34	3.622	-5.773	1.789
3	0.064	0.51	0.91	0.3	3.782	-5.77	1.799
4	0.065	0.23	0.34	0.14	3.412	-3.81	0.648
5	0.069	0.2	0.19	0.36	3.461	-3.916	1.896
6	0.07	0.68	0.52	0.34	3.508	-11.391	3.449
7	0.074	0.74	0.85	0.47	2.678	-7.475	2.314
8	0.075	0.63	0.7	0.1	3.309	-4.898	1.262
9	0.079	0.8	0.82	0.45	3.145	-10.817	3.464
10	0.084	0.72	0.56	0.44	3.037	-7.486	2.921
11	0.088	0.62	0.7	0.19	3.611	-13.507	4.389
12	0.096	0.12	0.84	0.39	3.343	-4.715	2.527
13	0.097	0.47	0.29	0.5	3.476	-5.73	2.211

14	0.098	0.36	0.25	0.44	3.342	-6.086	2.819
15	0.107	0.81	0.83	0.21	3.202	-13.665	4.678
16	0.113	0.14	0.18	0.44	3.417	-12.924	6.686
17	0.117	0.24	0.69	0.46	3.711	-8.246	3.813
18	0.118	0.37	0.75	0.38	3.379	-9.322	3.956
19	0.119	0.67	0.69	0.36	2.914	-8.048	3.231
20	0.119	0.85	0.75	0.33	2.907	-12.725	4.4
21	0.12	0.4	0.89	0.25	3.473	-7.327	2.877
22	0.123	0.28	0.2	0.42	3.597	-9.686	4.649
23	0.126	0.2	0.49	0.14	3.904	-5.345	2.174
24	0.141	0.51	0.67	0.27	3.705	-16.671	6.12
25	0.144	0.32	0.37	0.4	3.447	-9.482	3.965
26	0.146	0.48	0.67	0.22	3.954	-18.614	6.123
27	0.155	0.49	0.8	0.11	4.028	-13.866	4.13
28	0.156	0.26	0.26	0.42	3.47	-5.425	2.761
29	0.158	0.81	0.37	0.18	3.385	-10.853	3.354
30	0.16	0.67	0.54	0.44	3.255	-19.456	7.16

Supplementary References

1. Liu Z, *et al.* Data-driven inverse design of flexible pressure sensors. *PANS* **121**, e2320222121 (2024).
2. Yao G, *et al.* Bioinspired Triboelectric Nanogenerators as Self-Powered Electronic Skin for Robotic Tactile Sensing. *Adv. Funct. Mater.* **30**, 1907312 (2019).
3. Ha M, *et al.* Skin-Inspired Hierarchical Polymer Architectures with Gradient Stiffness for Spacer-Free, Ultrathin, and Highly Sensitive Triboelectric Sensors. *ACS Nano* **12**, 3964-3974 (2018).
4. Lee S, Park J-W. Fingerprint-inspired triboelectric nanogenerator with a geometrically asymmetric electrode design for a self-powered dynamic pressure sensor. *Nano Energy* **101**, 107546 (2022).
5. Zhong Y, *et al.* Dome-Conformal Electrode Strategy for Enhancing the Sensitivity of BaTiO3-Doped Flexible Self-powered Triboelectric Pressure Sensor. *ACS Appl. Mater. Interfaces* **16**, 1727-1736 (2023).
6. Si S, *et al.* 3D interlocked all-textile structured triboelectric pressure sensor for accurately measuring epidermal pulse waves in amphibious environments. *Nano Research*, **17**, 1923-1932 (2023).
7. Zu L, *et al.* Multiangle, self-powered sensor array for monitoring head impacts. *Sci. Adv.* **9**, eadg5152 (2023).
8. Shrestha K, *et al.* A Breathable, Reliable, and Flexible Siloxene Incorporated Porous SEBS-Based Triboelectric Nanogenerator for Human-Machine Interactions. *Adv. Energy Mater.* **14**, 2302471 (2023).
9. Zhu M, Wang Y, Lou M, Yu J, Li Z, Ding B. Bioinspired transparent and antibacterial electronic skin for sensitive tactile sensing. *Nano Energy* **81**, 105669 (2021).
10. Guo M, Xia Y, Liu J, Zhang Y, Li M, Wang X. Wearable Pressure Sensor Based on Triboelectric Nanogenerator for Information Encoding, Gesture Recognition, and Wireless Real-Time Robot Control. *Adv. Funct. Mater.* **35**, 2419209 (2025).
11. Chen SW, *et al.* Hierarchical elastomer tuned self-powered pressure sensor for wearable multifunctional cardiovascular electronics. *Nano Energy* **70**, 104460 (2020).
12. Hu H, *et al.* High Sensitivity Triboelectric Based Flexible Self-Powered Tactile Sensor with Bionic Fingerprint Ring Structure. *ACS. Sens.* **9**, 2907-2914 (2024).
13. Xie L, *et al.* Ultrasensitive Wearable Pressure Sensors with Stress-Concentrated Tip-Array Design for Long-Term Bimodal Identification. *Adv. Mater.* **36**, 2406235 (2024).
14. Qin S, *et al.* Triboelectric sensor with ultra-wide linear range based on water-containing elastomer and ion-rich interface. *Nat. Commun.* **15**, 10640 (2024).
15. Ren F, et al. Accelerated discovery of metallic glasses through iteration of machine learning and high-throughput experiments. Sci. Adv. 4, eaaq1566 (2018).
16. Chen T, et al. Machine intelligence-accelerated discovery of all-natural plastic substitutes. Nat. Nanotechnol. 19, 782-791 (2024).
17. Yang H, et al. Automatic strain sensor design via active learning and data augmentation for soft machines. Nature Mach. Intell. 4, 84-94 (2022).

18. Wang Z, Volinsky AA, Gallant ND. Crosslinking effect on polydimethylsiloxane elastic modulus measured by custom‐built compression instrument. *J. Appl. Polym. Sci.* **131**, 41050 (2014).
